# Metabolic Regulation of Phenanthrene-Degrading Strain *Sphingobium abikonense* GHP1 in Response to Phenanthrene Stress and Its Effects on Endorhizosphere Microbiota of Lettuce

**DOI:** 10.3390/plants15203136

**Published:** 2026-10-14

**Authors:** Lihang Zhang, Zhangkai Wang, Yizhen Jia, Ling’en Liu, Yi Yang, Weizheng Ren, Haiping Gu

**Affiliations:** College of Forestry, Henan Agricultural University, Zhengzhou 450046, China; zlihang06@163.com (L.Z.);

**Keywords:** phenanthrene, *Sphingobium abikonense*, untargeted metabolomics, lettuce, endophytic bacterial community

## Abstract

Polycyclic aromatic hydrocarbons (PAHs) are persistent organic pollutants with high ecotoxicity and bioaccumulation potential, and plant-microbe-assisted remediation is a promising green strategy for their removal. This study investigated *Sphingobium abikonense* GHP1, a phenanthrene-degrading bacterium isolated from a municipal solid waste incineration site, to elucidate its potential remediation mechanism using untargeted metabolomics and root endophytic bacterial community analysis. GHP1 completely degraded phenanthrene (50 mg L^−1^) within 3 days using it as the sole carbon source. During degradation, aromatic intermediates, including 1-hydroxy-2-naphthoic acid, salicylic acid, and hydroxybenzoic acid derivatives, accumulated, while central carbon metabolism, 2-oxocarboxylic acid metabolism, aromatic compound degradation, and benzoate degradation pathways were activated. Increased levels of NAD, purine metabolites, phosphatidylcholine, and phosphatidylethanolamine suggested enhanced redox regulation, energy metabolism, and membrane homeostasis under phenanthrene stress. In lettuce, GHP1 inoculation promoted plant growth, increased biomass and chlorophyll content, and reduced phenanthrene residues in roots and leaves. Moreover, GHP1 increased the richness of the root endophytic bacterial community and enriched Actinobacteriota, Firmicutes, and other bacterial taxa. These findings demonstrate that GHP1 enhances phenanthrene remediation through coordinated pollutant degradation, metabolic regulation, and plant-microbe interactions, providing a promising microbial resource for the remediation of PAH-contaminated environments.

## 1. Introduction

Polycyclic aromatic hydrocarbons (PAHs) are a class of persistent organic pollutants composed of two or more fused benzene rings, originating primarily from fossil fuel combustion, industrial activities, and vehicular emissions [1]. Owing to their strong hydrophobicity, high environmental persistence, and potential biological toxicity, PAHs have become important environmental contaminants of global concern. Phenanthrene (Phe) is a typical three-ring PAH that is widely distributed in soils, aquatic environments, and agricultural ecosystems. It can be taken up by plants and subsequently accumulate in plant tissues, thereby posing potential risks to ecological security and human health through food-chain transfer [2]. Phenanthrene has the molecular formula C_14_H_10_. Because of its relatively simple structure, widespread environmental occurrence, and representative transport and transformation characteristics of PAHs, phenanthrene is commonly used as a model compound for investigating the environmental fate and microbial degradation of PAHs [3].

Currently, the remediation of PAH-contaminated environments mainly relies on physical remediation, chemical oxidation, and bioremediation. Among these approaches, bioremediation has received considerable attention because of its low cost, limited environmental impact, and high sustainability [4]. Bacteria are widely distributed and highly diverse microorganisms with relatively rapid growth rates, and they represent one of the most extensively studied and widely applied groups of PAH-degrading microorganisms. Compared with other microbial groups, bacteria generally exhibit strong environmental adaptability, ease of cultivation and genetic manipulation, and high degradation potential [5]. Microbial degradation is an important pathway for the natural attenuation of PAHs. Previous studies have shown that PAH-degrading bacteria can catalyze the oxidation of aromatic rings through key enzyme systems, such as dioxygenases and monooxygenases, and subsequently convert PAHs into intermediate metabolites, including salicylic acid, phthalic acid, and catechol. These intermediates can then undergo ring-cleavage reactions and enter central carbon metabolic pathways, ultimately leading to mineralization [6].

To date, numerous bacterial strains capable of degrading PAHs have been isolated, particularly from genera such as *Pseudomonas*, *Sphingobium*, and *Rhodococcus*. Other genera, including *Achromobacter*, *Comamonas*, and *Mycobacterium*, have also been reported to degrade phenanthrene. For instance, *Achromobacter* sp. LH-1 efficiently degrades phenanthrene while enhancing its bioavailability through biosurfactant production [7], whereas *Achromobacter denitrificans* PheN1 is capable of nitrate-dependent anaerobic phenanthrene degradation [8]. *Comamonas testosteroni* can degrade phenanthrene and other PAHs through metabolic pathways that differ from those characterized in *Pseudomonas* [9]. Members of the family Sphingomonadaceae and its closely related taxa are also well known for their ability to metabolize PAHs and other aromatic compounds [10]. Among them, *Sphingobium* sp. SHPJ-2 exhibited a high capacity for degrading multiple PAHs [11], while *Sphingobium yanoikuyae* SJTF8 degraded 99% of phenanthrene at an initial concentration of 50 mg·L^−1^ within 48 h [12]. Collectively, these studies highlight *Sphingobium* as a promising microbial resource for the bioremediation of PAH-contaminated environments.

PAH-degrading bacteria can also interact with plants to enhance the remediation efficiency of contaminated environments. Some PAH-degrading bacteria can stably colonize the plant rhizosphere, while others can enter plant tissues and establish themselves as endophytic microorganisms [13]. Plant root exudates can provide carbon sources and energy for these bacteria, thereby promoting their growth and colonization. In turn, PAH-degrading bacteria can transform PAHs into the rhizosphere or plant tissues, reducing the bioavailability of pollutants and their accumulation within plants. For example, Li et al. [14] constructed a functional endophytic bacterial consortium and inoculated it into vegetables, demonstrating that endophytic bacterial colonization promoted PAH degradation and significantly reduced PAH accumulation in plant tissues. Zhao et al. [15] reported that the combined application of resveratrol and the PAH-degrading ryegrass endophyte C1 enhanced the removal of phenanthrene and naphthalene from contaminated soil and altered the microbial community structure in the contaminated environment. These findings indicate that PAH-degrading bacteria not only directly participate in the transformation of PAHs but may also enhance plant–microbe-assisted remediation by influencing plant growth, pollutant transport and translocation, and plant-associated microbial communities.

In the present study, *Sphingobium* abikonense GHP1 (*S. abikonense* GHP1), a phenanthrene-degrading bacterium isolated from a long-term municipal waste incineration site in Xinyang, Henan Province, China, was selected as the target microorganism. Previous studies have demonstrated that GHP1 can utilize phenanthrene as the sole carbon source, produce lipopeptide biosurfactants, and exhibit strong phenanthrene-degrading potential [16]. However, its metabolic responses and potential degradation pathways during phenanthrene degradation, as well as its effects on phenanthrene accumulation in plants and root-associated microbial communities, remain poorly understood. Therefore, untargeted metabolomics was first employed to characterize the metabolic responses of GHP1 during phenanthrene degradation and identify potential degradation intermediates and pathways. A lettuce pot experiment was subsequently conducted to evaluate the effects of GHP1 inoculation on plant growth and phenanthrene uptake, accumulation, and translocation, followed by root endophytic microbial community sequencing to assess changes in microbial composition, diversity, and potential functional profiles. Overall, this study aimed to elucidate the potential mechanisms by which GHP1 contributes to phenanthrene remediation from the perspectives of bacterial metabolism, plant phenanthrene accumulation, and root endophytic microbial community responses, providing a theoretical basis and microbial resource for the development of efficient and environmentally friendly PAH bioremediation strategies.

## 2. Results

### 2.1. Phenanthrene Degradation by Strain GHP1 in Liquid Medium

**Figure 1:**
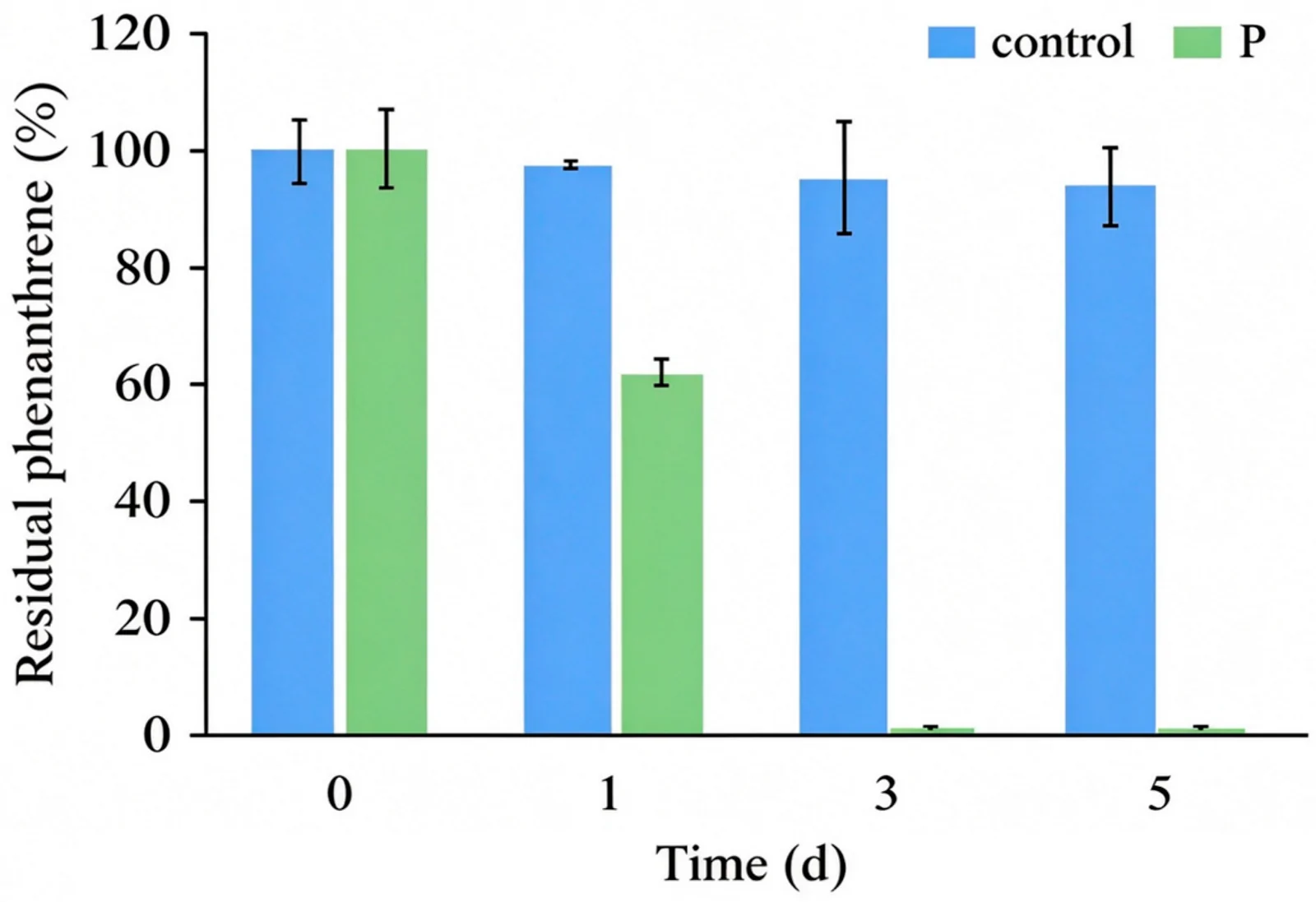
Dynamics of phenanthrene residual rate during degradation by strain GHP1.

The changes in the residual phenanthrene concentration over time in the liquid mineral medium containing phenanthrene as the sole carbon source (initial concentration, 50 mg·L^−1^) are shown in Figure 1. In the uninoculated control, the residual phenanthrene concentration remained relatively high throughout the incubation period, indicating that abiotic losses of phenanthrene were negligible under the experimental conditions. In contrast, the residual phenanthrene concentration decreased rapidly following inoculation with strain GHP1 (P), and phenanthrene was almost completely degraded by day 3, demonstrating the strong phenanthrene-degrading capacity of GHP1. Kinetic fitting showed that phenanthrene degradation by GHP1 followed a first-order kinetic model (*R*^2^ = 0.99), with a degradation rate constant (*k*) of 1.125 d^−1^ and a half-life of 0.616 d (Table 1). These results demonstrate that strain GHP1 can efficiently promote the degradation of phenanthrene in liquid medium.

### 2.2. Metabolomic Analysis of Strain GHP1 During Phenanthrene Degradation

#### 2.2.1. Differential Metabolites During Phenanthrene Degradation by GHP1

**Figure 2:**
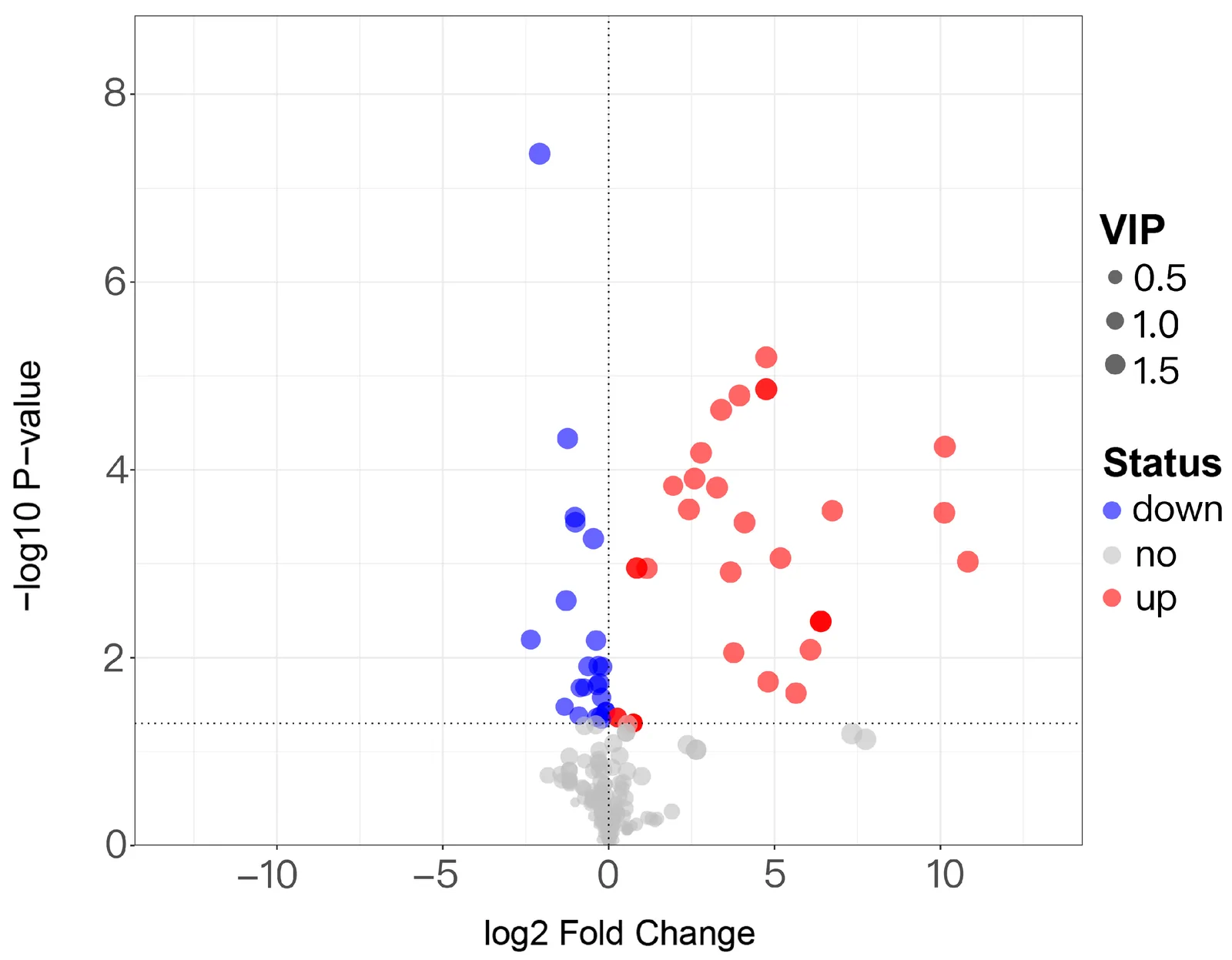
Volcano plot comparison between P group and control. NEG mode at day 1.

Based on the phenanthrene degradation profile of strain GHP1 described above, untargeted metabolomic analysis was performed on samples collected on days 0, 1, and 3 of the phenanthrene degradation process. The volcano plots (Figure 2, Figure S1) showed that, in the negative ion mode (NEG), a total of 97 metabolites were upregulated on day 0, including 1-hydroxy-2-naphthoic acid and 3-phenylpropanoic acid, whereas six metabolites were downregulated, including 3-hydroxyglutaric acid. On day 1, 33 metabolites, including 1-hydroxy-2-naphthoic acid, salicylic acid, and uridine, were upregulated, whereas 23 metabolites, including trans-cinnamic acid and 8-oxononanoic acid, were downregulated. Notably, 3-hydroxyglutaric acid showed significant downregulation from day 0 to day 3 (*p* < 0.05). By day 3, 29 metabolites, including pyruvate, citraconic acid, and monomethyl fumarate, were upregulated, whereas trans-cinnamic acid and 4-vinylphenol were downregulated.

In the positive ion mode (POS), 87 metabolites were upregulated on day 0, including nicotinamide adenine dinucleotide (NAD), adenosine, and cytosine, whereas 12 metabolites, including maltose and trehalose, were downregulated. On day 1, 25 metabolites, including adenosine and hypoxanthine, were upregulated, whereas 23 metabolites, including 1-palmitoylglycerol and methyl ricinoleate, were downregulated. By day 3, 23 metabolites, including phosphatidylcholine (PC), phosphatidylethanolamine (PE), and adenine, were upregulated.

#### 2.2.2. Hierarchical Clustering Analysis of Differential Metabolites During Phenanthrene Degradation

**Figure 3:**
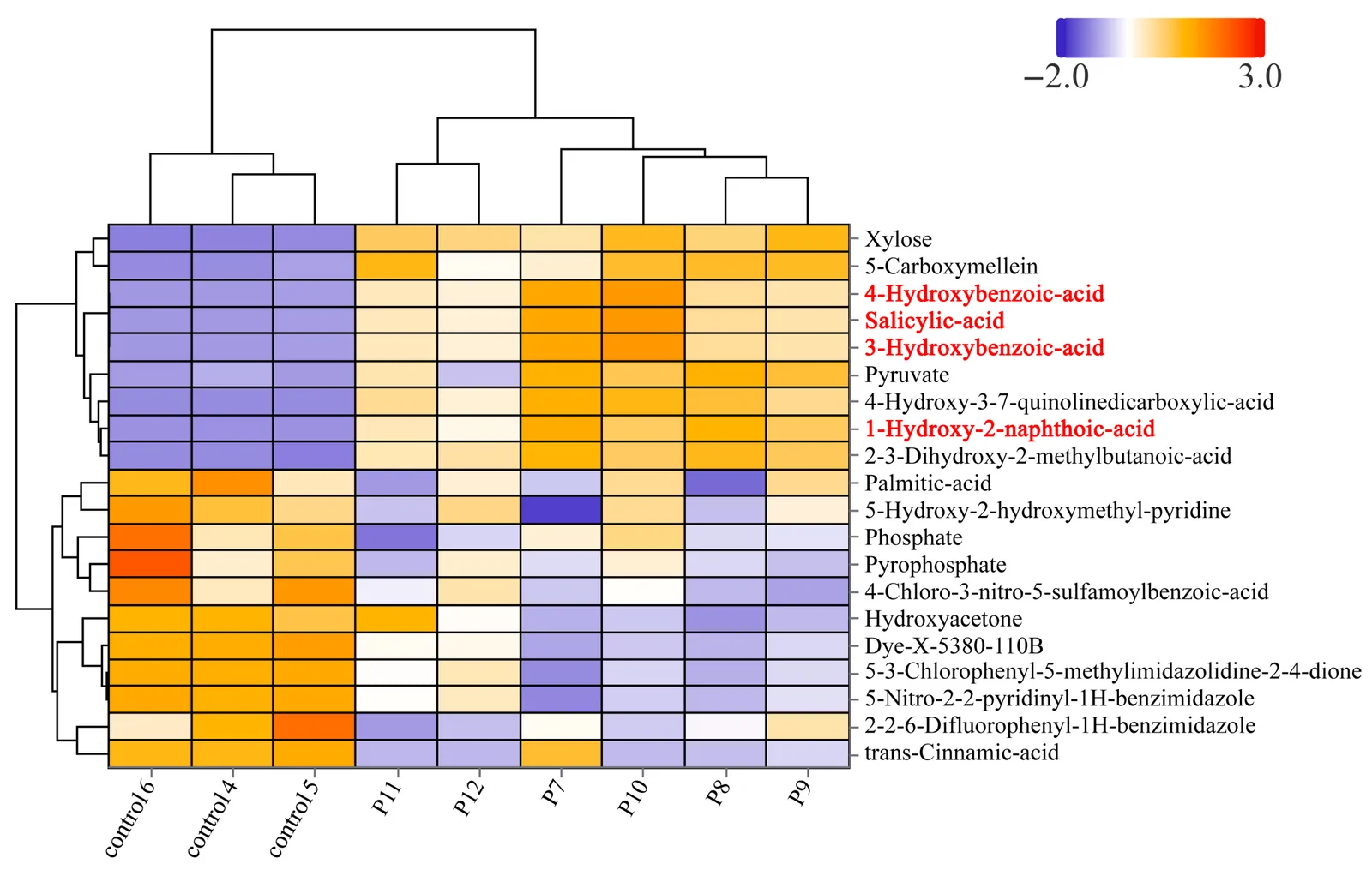
Heatmap of differential metabolites in P vs. Control. NEG mode at day 1. Metabolites marked in red indicate the key substances mentioned in the text.

In the negative ion mode (NEG), 1-hydroxy-2-naphthoic acid was already detected in the P group on day 0, with a higher abundance than that observed in the control (Figure S2). On day 1, the abundances of several key intermediate metabolites associated with phenanthrene degradation, including 1-hydroxy-2-naphthoic acid, salicylic acid, 3-hydroxybenzoic acid, and 4-hydroxybenzoic acid, were increased in the P group (Figure 3). These results indicate that GHP1 rapidly produced and accumulated multiple aromatic degradation intermediates during the early stage of phenanthrene degradation. By day 3, the abundances of metabolites associated with central carbon metabolism, such as pyruvate and succinic anhydride, were further increased, suggesting that phenanthrene degradation intermediates may have progressively entered downstream carbon metabolic processes.

In the positive ion mode (POS), the abundances of carbohydrate-related metabolites, including maltose, trehalose, melibiose, and sucrose, were lower in the P group than in the control on day 0, suggesting that GHP1 may have undergone adjustments in carbon and energy metabolism during the initial stage of phenanthrene exposure. On day 1, the abundances of nucleosides and nitrogen metabolism-related metabolites, including cytosine and adenosine, were higher in the P group than in the control. By day 3, multiple metabolites in the P group showed an overall increasing trend. In particular, the abundances of purine- and pyrimidine-related metabolites, including adenosine, cytosine, hypoxanthine, and adenine, were higher in the P group than in the control. Meanwhile, the abundances of metabolites associated with membrane lipid biosynthesis, such as 1,2-dipalmitoleoyl-sn-glycero-3-phosphocholine and phosphatidylethanolamine (18:0/18:2), also increased. These results indicate that nucleotide metabolism and membrane lipid metabolism in GHP1 underwent changes as phenanthrene degradation progressed.

#### 2.2.3. Correlation Analysis of Metabolites Associated with Phenanthrene Degradation by GHP1

**Figure 4:**
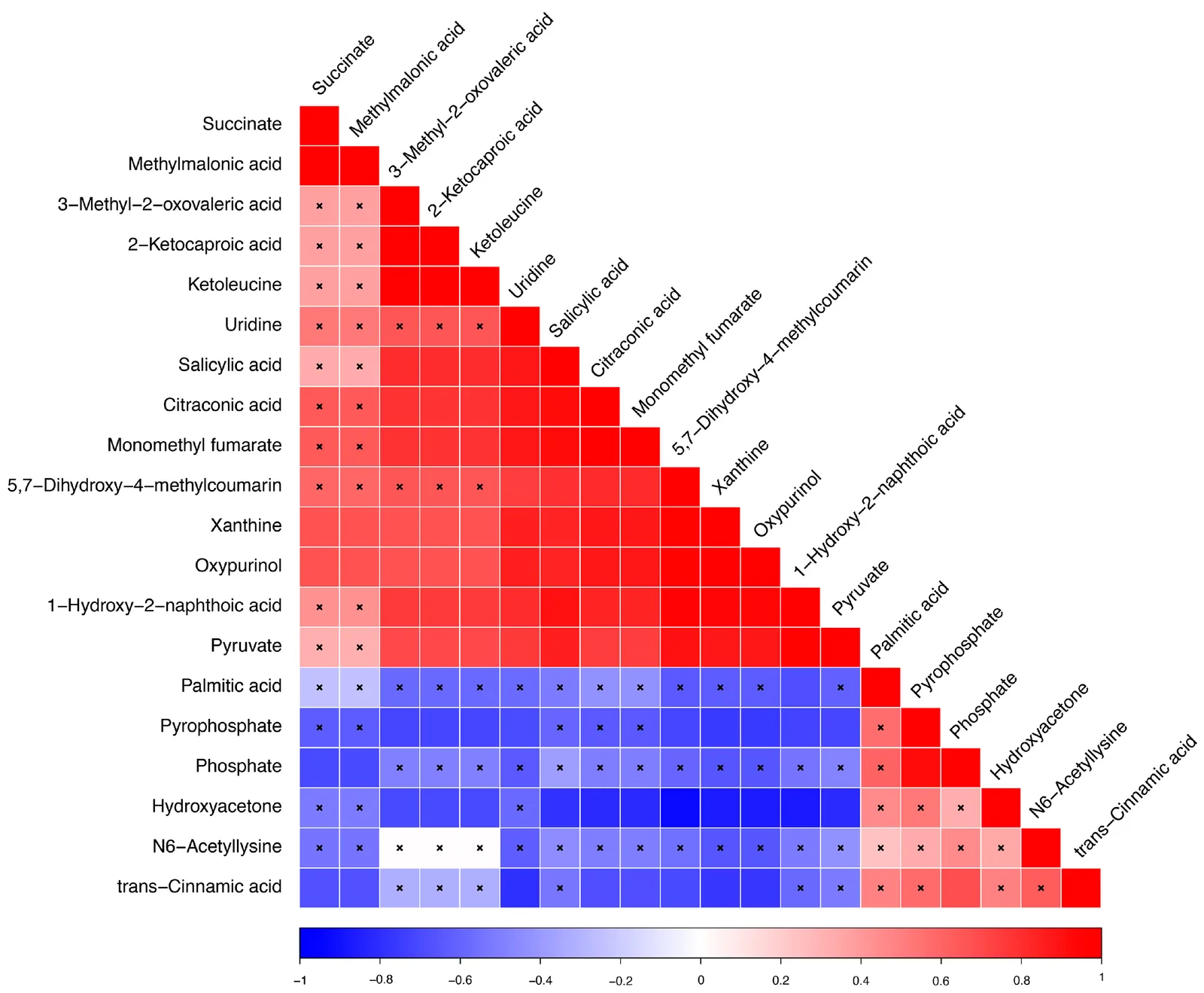
Correlation analysis of differential metabolites in P vs. Control. NEG mode at day 1. Symbols marked with “×” indicate correlations with |r| < 0.6.

In the negative ion mode (NEG), correlation analysis between the P and control on day 0 showed that most differential metabolites were positively correlated, with relatively high correlation coefficients, and most r values exceeded 0.60 (Figure 4, Figure S3). Among them, 1-hydroxy-2-naphthoic acid showed the strongest positive correlations with pyruvate (*r* = 0.936), isovaleric acid (*r* = 0.932), and valeric acid (*r* = 0.932). On day 1, 1-hydroxy-2-naphthoic acid was positively correlated with pyruvate (*r* = 0.967), xanthine (*r* = 0.947), oxypurinol (*r* = 0.947), and salicylic acid (*r* = 0.901), indicating coordinated changes between key phenanthrene degradation intermediates and metabolites involved in central carbon and purine metabolism. In contrast, 1-hydroxy-2-naphthoic acid showed negative correlations with hydroxyacetone (*r* = −0.887) and pyrophosphate (*r* = −0.723), suggesting that some metabolites underwent opposite dynamic changes during phenanthrene degradation. In addition, salicylic acid was strongly positively correlated with citraconic acid (*r* = 0.920), further suggesting a potential metabolic association between aromatic degradation intermediates and downstream organic acid metabolism. On day 3, uridine, xanthine, and oxypurinol were highly positively correlated with each other (*r* > 0.91) and exhibited coordinated variation with pyruvate, 3-hydroxyvaleric acid, and xylose. Across all three sampling time points, the three branched-chain keto acids, 2-oxohexanoic acid, 4-methyl-2-oxopentanoic acid, and 3-methyl-2-oxopentanoic acid, showed a completely positive correlation with each other (*r* = 1.00). On day 3, these metabolites also exhibited highly synchronized changes with pyruvate and 4-oxohexanoic acid (*r* > 0.95), suggesting strong metabolic coupling among branched-chain amino acid metabolism, 2-oxoacid metabolism, and central carbon metabolism during the later stage of phenanthrene degradation.

In the positive ion mode (POS), maltose, trehalose, and sucrose showed perfect positive correlations with each other on day 0 (*r* = 1.00), indicating consistent changes in these carbohydrate metabolites during the initial stage of phenanthrene stress. Adenosine was negatively correlated with maltose and other carbohydrate metabolites, indicating that increases in adenosine levels were accompanied by decreases in the abundance of carbohydrates and osmoprotective metabolites. This pattern may reflect adjustments in the synthesis or utilization of reserve carbohydrates during the initiation of phenanthrene degradation and associated metabolic responses. On day 1, adenosine, nicotinamide adenine dinucleotide (NAD), cytosine, and phospholipid metabolites, including phosphatidylcholine (PC) and phosphatidylethanolamine (PE), showed strong positive correlations (*r* > 0.95), suggesting coordinated responses among energy metabolism, redox regulation, nucleotide metabolism, and membrane lipid metabolism. By day 3, adenosine, NAD, cytosine, arecaidine, and multiple phospholipid metabolites, including PC and PE, remained highly positively correlated (*r* > 0.95). These results suggest that the metabolic network of GHP1 became increasingly coordinated as phenanthrene degradation progressed, with redox metabolism, nucleotide metabolism, and membrane lipid metabolism potentially contributing collectively to bacterial adaptation to phenanthrene stress and its degradation.

#### 2.2.4. KEGG Enrichment Analysis of GHP1 During Phenanthrene Degradation

**Figure 5:**
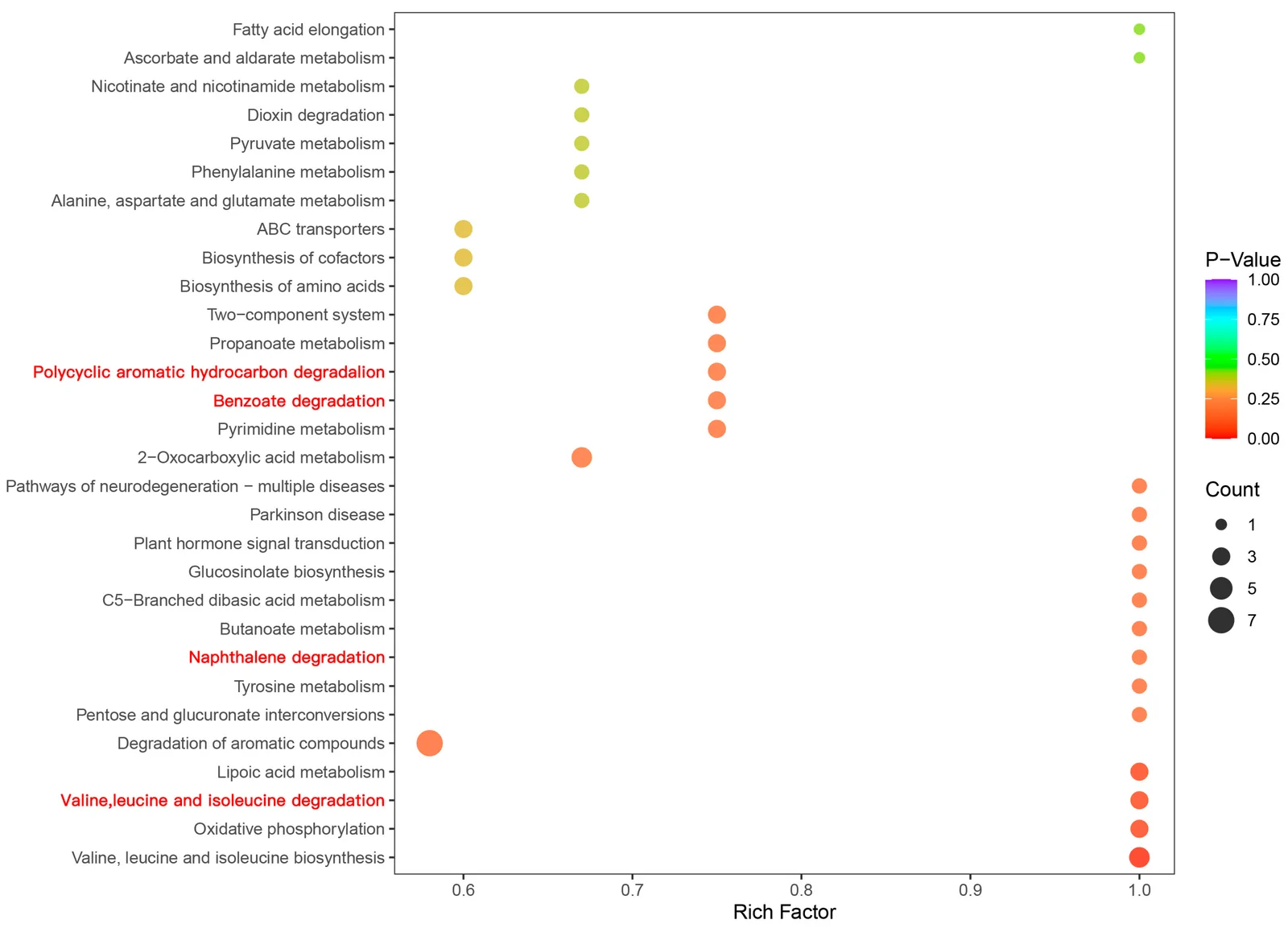
KEGG enrichment plot of differential metabolites between P group and control. NEG mode at day 1. Metabolites marked in red indicate the key substances mentioned in the text.

In the negative ion mode (NEG), KEGG enrichment analysis comparing the P and control groups (Figure 5, Figure S4) showed that the valine, leucine, and isoleucine degradation pathway exhibited a near-significant enrichment trend at different incubation times, with the strongest trend observed on day 1 (*p* = 0.065). This finding suggests that branched-chain amino acid metabolism may be involved in the response of GHP1 to phenanthrene stress and subsequent regulation of energy metabolism. On day 0, several pathways associated with aromatic pollutant degradation and substance transport were detected in the P group, including polycyclic aromatic hydrocarbon degradation (ko00624), degradation of aromatic compounds (ko01220), naphthalene degradation (ko00626), benzoate degradation (ko00362), and ABC transporters (ko02010).

However, none of these pathways reached statistically significant enrichment. On day 1, the number of enriched metabolites associated with the PAH degradation pathway decreased from four on day 0 to three, while the number associated with aromatic compound degradation decreased from nine to seven (*p* = 0.155). The enrichment of the naphthalene degradation pathway increased relative to day 0 (*p* = 0.168), whereas the benzoate degradation pathway showed little change (*p* = 0.550). These results suggest that GHP1 had initiated metabolic responses associated with aromatic compound degradation during the early stage of phenanthrene degradation, although the enrichment of these pathways did not reach statistical significance. By day 3, the 2-oxocarboxylic acid metabolism pathway (ko01210, *p* = 0.013) exhibited an enrichment trend, suggesting that some aromatic degradation intermediates may have been further converted into intermediates associated with 2-oxocarboxylic acid metabolism and subsequently incorporated into central carbon metabolism for energy production. In addition, nitrotoluene degradation (ko00633), phenylalanine metabolism (ko00360, *p* = 0.106), and xylene degradation (ko00622, *p* = 0.208) showed weak enrichment trends, indicating that the later stage of phenanthrene degradation may involve coordinated responses of multiple pathways associated with aromatic compound metabolism. Furthermore, the pentose and glucuronate interconversions pathway showed a trend toward enrichment (*p* = 0.040). This pathway is closely associated with pentose metabolism, carbon utilization, and the biosynthesis of nucleotide and cofactor precursors, and may contribute to supporting bacterial growth and enhancing the survival and adaptation of GHP1 under phenanthrene-contaminated conditions.

In the positive ion mode (POS), starch and sucrose metabolism (ko00500, *p* = 0.0336) and the phosphotransferase system (ko02060, *p* = 0.0336) presented an enrichment trend on day 0, while galactose metabolism (ko00052, *p* = 0.110) showed an enrichment trend. The ABC transporter pathway (ko02010) was also detected. These results suggest that, during the initial stage of phenanthrene stress, GHP1 may regulate carbohydrate metabolism, carbon-source uptake, and transmembrane transport to meet its metabolic requirements under phenanthrene exposure. Purine metabolism (ko00230) remained enriched on days 1 and 3. On day 1, this pathway showed an enrichment trend (*p* = 0.0889), whereas by day 3, the number of enriched metabolites increased from two to three and the *p* value decreased to 0.0114, reaching a statistically significant level of enrichment. Purine metabolism is closely associated with energy metabolism, nucleic acid biosynthesis, and cellular proliferation. Therefore, these results suggest that, as phenanthrene degradation progressed, GHP1 may have enhanced nucleotide and energy metabolism to support bacterial growth and continued pollutant degradation.

#### 2.2.5. Mechanism of Phenanthrene Degradation by Strain GHP1

**Figure 6:**
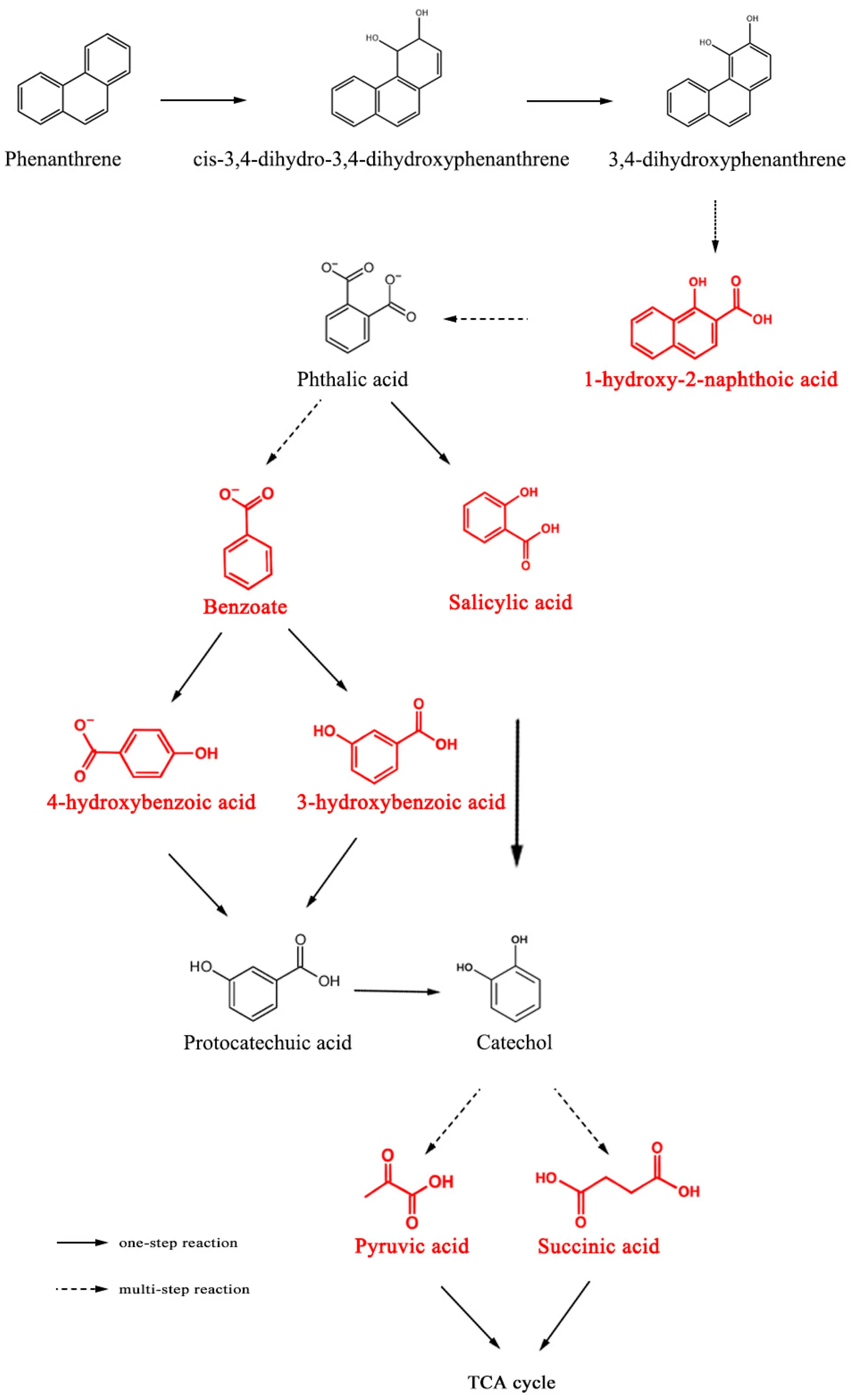
Putative phenanthrene degradation pathway of strain GHP1. Red: metabolites detected in this study; black: intermediates inferred from literature.

Based on the intermediate metabolites detected during phenanthrene degradation and the results of KEGG pathway enrichment analysis, it was proposed that strain GHP1 may degrade and transform phenanthrene through two complementary pathways, namely the phthalate and salicylate pathways (Figure 6). Phenanthrene may first be oxidized by relevant oxygenase systems to form key intermediates such as 1-hydroxy-2-naphthoic acid, followed by its conversion to phthalate. The resulting intermediates may then undergo further degradation through two possible routes. On the one hand, phthalate may enter the phthalate degradation pathway and subsequently generate aromatic intermediates such as 4-hydroxybenzoate and 3-hydroxybenzoate. On the other hand, phenanthrene-derived intermediates may be converted to salicylate and subsequently enter downstream aromatic compound degradation pathways.

Both pathways may ultimately converge at central aromatic degradation intermediates such as catechol, which can undergo ring-cleavage reactions to generate small organic acids, including pyruvate and succinate. These metabolites can subsequently enter the tricarboxylic acid (TCA) cycle and other central carbon metabolic pathways, thereby facilitating the further transformation and utilization of phenanthrene-derived carbon. Collectively, these results suggest that GHP1 may employ a branched metabolic network involving aromatic ring oxidation, formation and transformation of aromatic intermediates, ring cleavage, and subsequent integration into central carbon metabolism during phenanthrene degradation.

### 2.3. Effects of Phenanthrene Stress and Strain GHP1 Inoculation on Lettuce

#### 2.3.1. Responses of Lettuce Growth and Physiology to Phenanthrene Stress

**Figure 7:**
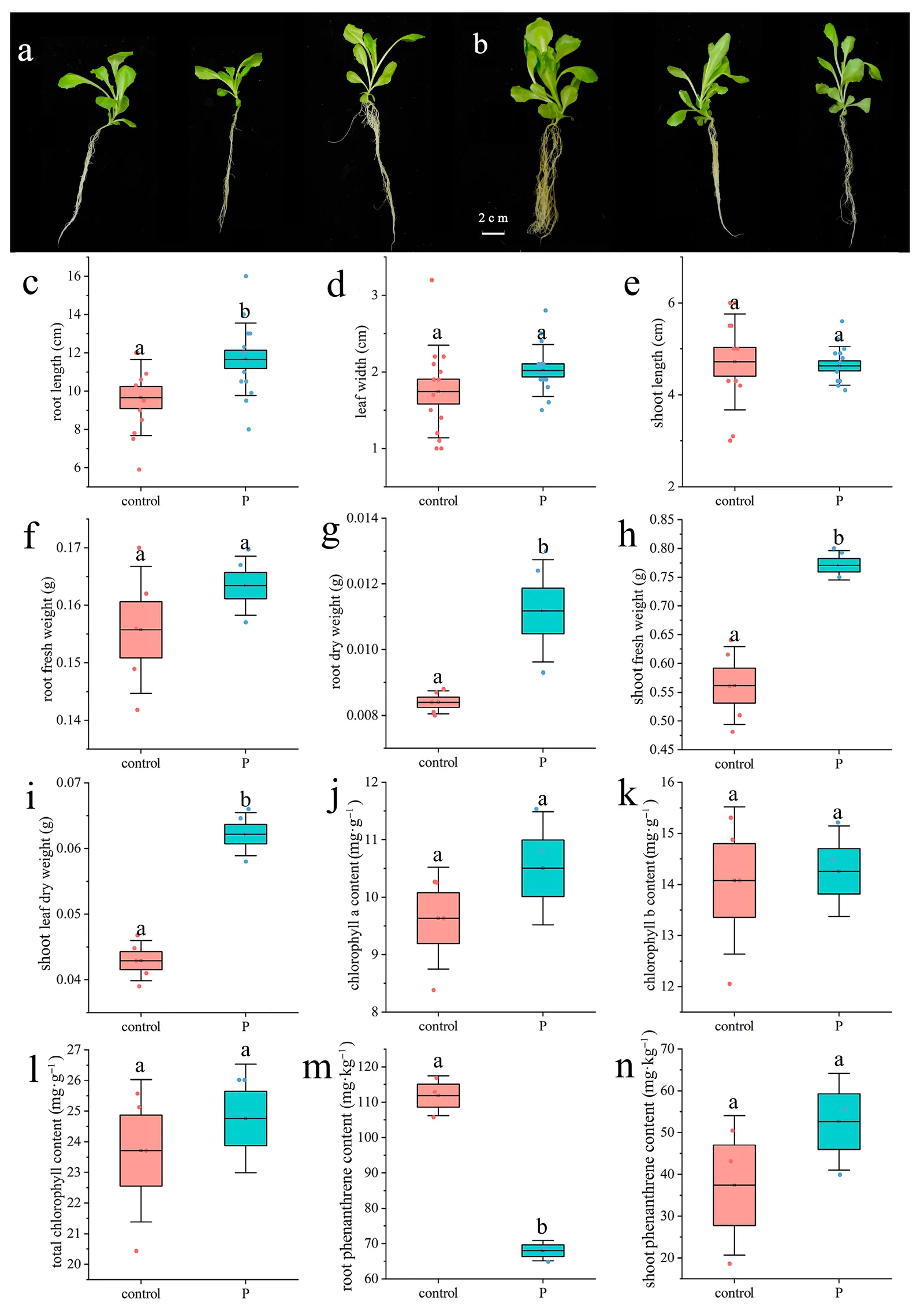
Impacts of strain GHP1 on lettuce growth and physiology under phenanthrene stress. Growth performance (**a**,**b**), root length (**c**), leaf width (**d**), shoot length (**e**), root fresh weight (**f**), root dry weight (**g**), shoot fresh weight (**h**), shoot dry weight (**i**), chlorophyll a (**j**), chlorophyll b (**k**) contents, total chlorophyll content (**l**), root phenanthrene content (**m**) and shoot phenanthrene content (**n**). Different lowercase letters indicate statistically significant differences (*p* < 0.05).

After 42 days of cultivation in MS medium supplemented with 50 mg·kg^−1^ phenanthrene, lettuce plants inoculated with GHP1 (P group) exhibited better overall growth than those in the control, as evidenced by more developed root systems, a greater number of lateral roots, and denser lateral root distribution (Figure 7b). In addition, compared with the control, the P group showed increases in root length (Figure 7c), leaf width (Figure 7d), and shoot length (Figure 7e), indicating that inoculation with strain GHP1 alleviated the inhibitory effects of phenanthrene stress on lettuce growth. In terms of biomass accumulation, GHP1 inoculation also increased the root fresh weight (Figure 7f), root dry weight (Figure 7g), shoot fresh weight (Figure 7h), and shoot dry weight (Figure 7i) of lettuce. Furthermore, the contents of chlorophyll a and chlorophyll b were significantly higher in the P group than in the control, suggesting that GHP1 inoculation helped maintain leaf photosynthetic pigment levels and thereby improved the physiological status of lettuce under phenanthrene stress.

The analysis of residual phenanthrene concentrations (Table 2) showed that phenanthrene accumulation differed between lettuce roots and shoots. The phenanthrene concentration in the roots decreased significantly from 118.83 mg·kg^−1^ in the control treatment to 33.23 mg·kg^−1^ in the P treatment (*p* = 0.0013). In contrast, no significant difference was observed in the shoots, where phenanthrene concentrations were 46.79 and 52.61 mg·kg^−1^ in the control and P treatments, respectively (*p* = 0.66). These results indicate that inoculation with GHP1 significantly reduced phenanthrene accumulation in lettuce roots (Figure 7), possibly because strain GHP1 promoted the degradation of phenanthrene accumulated in the roots, thereby alleviating the inhibitory effects of phenanthrene on root growth and contributing to the activation of plant defense responses [17].

#### 2.3.2. Effects of Strain GHP1 Inoculation on the Endophytic Bacterial Community in Lettuce Roots

The alpha diversity indices (Sobs, Chao1, ACE, and Simpson) of the endophytic bacterial communities in lettuce roots of the control and P groups are presented in Table 3. Compared with the control, the P group showed a slight increase in species richness indices, whereas the Simpson index decreased. These results suggest that GHP1 inoculation may increase the richness of the endophytic bacterial community in lettuce roots to some extent, while potentially altering community evenness and the distribution of dominant bacterial taxa.

**Figure 8:**
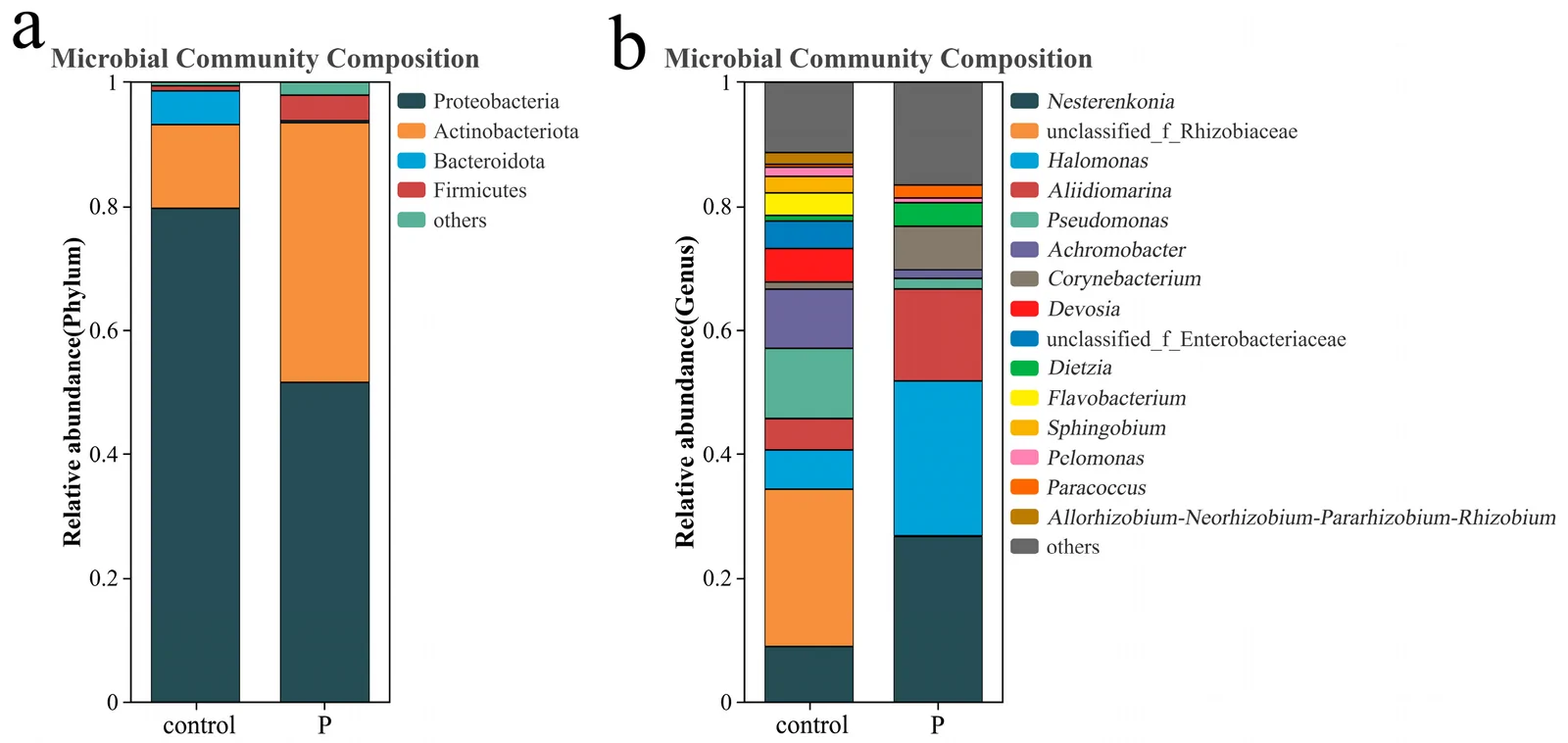
Bacterial community structure in lettuce roots under Phenanthrene stress at phylum (**a**) and genus (**b**) levels.

At the phylum level, the endophytic bacterial community in the roots of the control was predominantly composed of Pseudomonadota (Figure 8a). In contrast, the relative abundances of Actinobacteriota and Firmicutes increased in the P group, whereas that of Bacteroidota decreased, indicating that GHP1 inoculation altered the phylum-level composition of the endophytic bacterial community in lettuce roots. At the genus level (Figure 8b), unclassified_f_*Rhizobiaceae* was the most abundant taxon in the control, followed by *Pseudomonas* and *Achromobacter*. Compared with the control, the relative abundances of *Nesterenkonia*, *Aliidiomarina*, and *Halomonas* were increased in the P group, suggesting that GHP1 inoculation may promote the enrichment of certain bacterial taxa with enhanced tolerance or environmental adaptability in lettuce roots.

**Figure 9:**
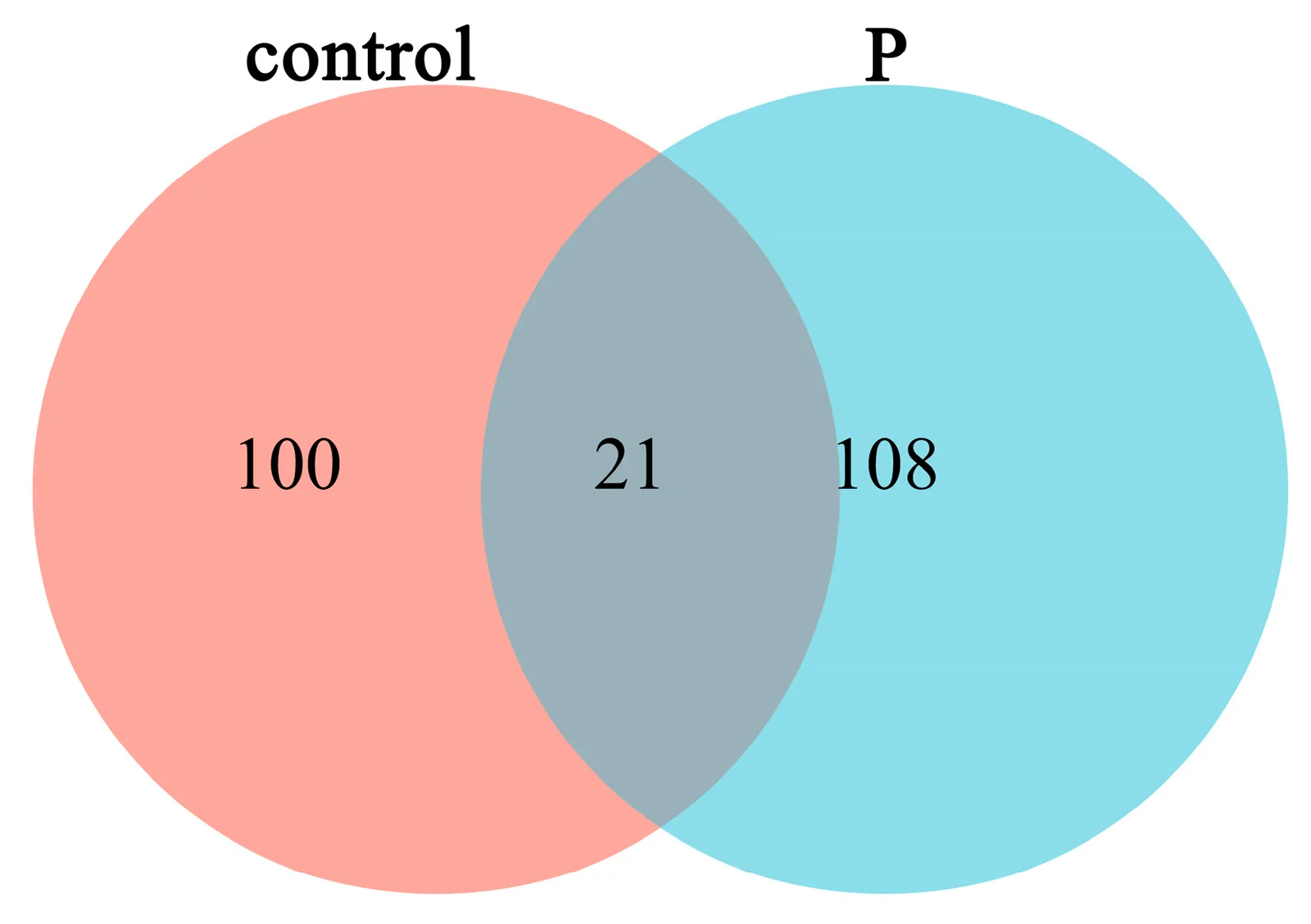
Venn diagram of endophytic bacterial communities in lettuce roots.

Venn diagram analysis (Figure 9) showed that 21 core OTUs were shared between the control and P groups, suggesting that these OTUs may represent relatively stable and resident microbial taxa in lettuce roots. The P group contained 108 unique OTUs, slightly higher than the 100 unique OTUs detected in the control group. These results indicate that GHP1 inoculation exerted a certain influence on the endophytic microbial community of lettuce roots and may have promoted the enrichment of specific endophytic bacterial taxa, thereby increasing differences in community composition and potentially enhancing its complexity.

**Figure 10:**
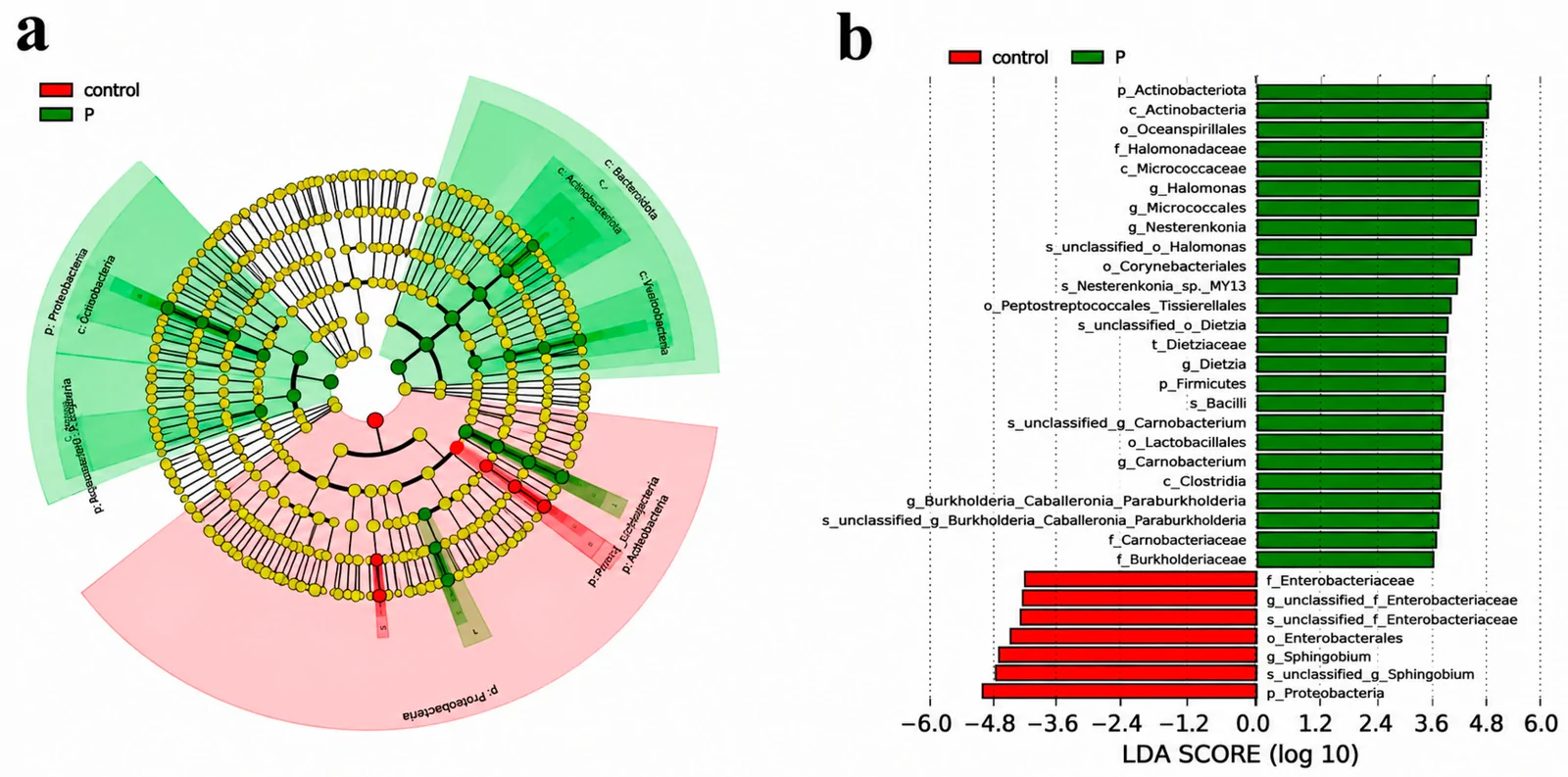
Phylogenetic tree and LDA score. (**a**) Cladogram showing the phylogenetic distribution of differentially abundant bacterial taxa. Circles from the centre outwards represent taxonomic ranks from kingdom to species. Red nodes indicate taxa enriched in the control group, green nodes represent taxa enriched in the P treatment group, and yellow nodes indicate taxa with no significant difference between groups. (**b**) LDA score histogram displaying taxa with significant differential abundance (LDA score > 1.0). Positive LDA scores correspond to taxa enriched in the P group (green bars), whereas negative LDA scores indicate taxa enriched in the control group (red bars). Prefixes before taxon names denote taxonomic levels: p_, phylum; c_, class; o_, order; f_, family; g_, genus; s_, species.

LEfSe cladogram analysis (Figure 10) showed that the characteristic taxa in the control group mainly included *Sphingobium* and Enterobacteriaceae, indicating that these taxa had relatively high abundances under phenanthrene stress in the absence of GHP1 inoculation. In contrast, the differential taxa in the P group were more widely distributed and were mainly affiliated with Actinobacteriota and Pseudomonadota, including *Dietzia, Nesterenkonia*, *Carnobacterium*, *Burkholderia*, and *Halomonas*. These results suggest that GHP1 inoculation was closely associated with changes in the composition of the bacterial community in lettuce roots.

Notably, although GHP1 belongs to the genus *Sphingobium*, this genus did not show an enrichment trend in the P group but instead showed relatively high abundance in the control. This finding does not necessarily indicate that GHP1 failed to colonize the lettuce roots. Because 16S rRNA gene amplicon sequencing generally has limited resolution at the strain level, it is difficult to distinguish the exogenously inoculated GHP1 strain from indigenous *Sphingobium* strains already present in the plant root-associated microbiota. The enrichment of *Sphingobium* in the control may therefore mainly reflect indigenous populations originating from the soil, seeds, or resident endophytic microbiota. Meanwhile, the increased abundance of other bacterial taxa in the P group may have reduced the relative proportion of *Sphingobium* within the overall bacterial community. Taken together, these results indicate that GHP1 inoculation markedly influenced the structure of the endophytic bacterial community in lettuce roots and promoted the enrichment of multiple functionally relevant or environmentally adaptable bacterial taxa.

## 3. Discussion

### 3.1. Metabolic Regulatory Mechanisms of Strain GHP1 in Response to Phenanthrene Stress

*Sphingobium abikonense* is a Gram-negative, aerobic bacterium belonging to the genus Sphingobium. The type strain (NBRC 16140^T^) was originally isolated from petroleum-contaminated soil, and members of this species have been reported to transform or degrade various persistent organic pollutants, including hexachlorocyclohexane, phenanthrene, and pentachlorophenol [18]. In the present study, *S. abikonense* GHP1 was isolated from a long-term municipal solid waste incineration site. In addition to evaluating its phenanthrene-degrading capacity, untargeted metabolomics was employed to characterize its metabolic responses to phenanthrene exposure and to investigate the potential metabolic basis underlying phenanthrene degradation.

Previous studies on *S. abikonense* have mainly focused on its pollutant-degrading performance under contaminated environmental conditions. For example, biochar-immobilized *S. abikonense* exhibited effective phenanthrene removal in phenanthrene-Cu co-contaminated soil, with substantial phenanthrene degradation occurring within six days [19]. Strong phenanthrene-degrading capacity has also been reported for other members of the genus *Sphingobium*. For instance, at an initial concentration of 50 mg·L^−1^, *Sphingobium yanoikuyae* SJTF8 achieved 99% degradation of phenanthrene within 48 h [12]. In contrast to these studies, which primarily emphasized pollutant degradation efficiency, the present study integrates phenanthrene degradation assessment with untargeted metabolomic analysis and plant-based experiments. Specifically, we further evaluated the effects of GHP1 inoculation on lettuce growth and the root endophytic bacterial community. This integrated approach provides additional insight into both the metabolic basis of phenanthrene degradation by GHP1 and its potential role in plant-microbe-assisted remediation of phenanthrene-contaminated environments.

In this study, untargeted metabolomic analysis was employed to elucidate the metabolic response characteristics of *Sphingobium abikonense* GHP1 under phenanthrene stress. The results showed a significant accumulation of 1-hydroxy-2-naphthoic acid immediately after GHP1 was exposed to phenanthrene (Figure 1). 1-Hydroxy-2-naphthoic acid is an important intermediate metabolite generated during phenanthrene degradation through dioxygenase-catalyzed reactions, and its formation is generally considered an important indicator of the initiation of the initial oxidation of the aromatic ring of phenanthrene [20,21]. Therefore, these findings suggest that GHP1 can rapidly respond to phenanthrene stress and initiate oxidative degradation processes targeting aromatic pollutants. Similarly, Puntus et al. [22] detected the accumulation of 1-hydroxy-2-naphthoic acid during phenanthrene degradation by strain BS3702 (pBS1143), further supporting its role as a characteristic intermediate metabolite in the microbial degradation of phenanthrene.

Meanwhile, the decreased level of 3-hydroxyglutaric acid on day 0 (Figure S1a) may reflect a metabolic reprogramming of carbon metabolism in GHP1 during the early stage of phenanthrene stress. As phenanthrene enters the cells as a novel exogenous carbon source, it must undergo a series of oxidation, ring-cleavage, and conversion reactions before being incorporated into central metabolic pathways. Therefore, GHP1 may adjust the distribution of pre-existing carbon metabolic fluxes to meet the requirements for the biosynthesis of enzymes involved in aromatic compound degradation and for energy supply [23].

Under the positive ion mode (POS), nitrogen-containing metabolites, including NAD, adenosine, and cytosine, were increased, suggesting enhanced redox regulation, energy metabolism, and nucleotide metabolism in GHP1 during phenanthrene degradation (Figure S1c). NAD^+^/NADH is an important redox cofactor involved in electron transfer during dioxygenase- and monooxygenase-catalyzed reactions and can provide reducing power for the hydroxylation of aromatic compounds, making it a key metabolic factor in the microbial degradation of PAHs [24]. Previous studies have shown that PAH-degrading bacteria may enhance nicotinamide-associated metabolism under pollutant stress to maintain cellular redox homeostasis and support the activity of degradation-related enzymes [25]. Therefore, the increase in NAD-related metabolites observed in this study may indicate an enhanced capacity for electron supply during phenanthrene oxidative degradation in GHP1.

In addition, the increased levels of adenosine and cytosine may be associated with increased nucleotide biosynthetic requirements during bacterial growth, suggesting that GHP1 maintained relatively high metabolic activity while utilizing phenanthrene as a carbon source. In contrast, the levels of carbohydrates such as maltose, trehalose, and sucrose decreased, potentially indicating an adjustment in carbon utilization strategies under phenanthrene stress. Trehalose is an important osmoprotective compound in microorganisms and plays a protective role in maintaining cellular structures under environmental stress [26]. The decrease in trehalose levels may be associated with the redistribution of carbon metabolism under phenanthrene stress, suggesting that GHP1 may regulate the utilization of stored carbon reserves and energy metabolism to maintain its environmental adaptability.

On day 1, aromatic metabolites, including 1-hydroxy-2-naphthoic acid, salicylic acid, 3-hydroxybenzoic acid, and 4-hydroxybenzoic acid, were increased in the P group (Figure 2), suggesting that the initial oxidation products of phenanthrene underwent further side-chain cleavage and aromatic ring transformation [27]. Salicylic acid and hydroxybenzoic acid derivatives are generally considered important downstream intermediates in phenanthrene degradation and can be further converted into catechol or protocatechuate, which subsequently enter the central metabolic network through ortho- or meta-cleavage pathways [28]. In addition, 1-hydroxy-2-naphthoic acid showed a strong positive correlation with pyruvate (Figure 3), indicating a coordinated relationship between the dynamics of the initial oxidation products of phenanthrene and enhanced central carbon metabolism. This finding suggests that aromatic degradation products may be progressively incorporated into the central metabolic network of GHP1 [29].

On day 3, the levels of pyruvate and succinate-related metabolites increased (Figure S2b), accompanied by significant enrichment of the 2-oxoacid metabolism pathway (Figure 4). These results suggest that small-molecule aromatic intermediates generated during phenanthrene degradation may be further incorporated into central carbon metabolic pathways, thereby providing energy and biosynthetic precursors for bacterial growth. Meanwhile, purine metabolism was enhanced, with increased levels of adenosine, adenine, and hypoxanthine (Figure S4b), further indicating that GHP1 maintained active energy metabolism and nucleotide biosynthesis during phenanthrene utilization, which may facilitate bacterial growth and adaptation to phenanthrene stress [30]. In addition, membrane phospholipids such as phosphatidylcholine (PC) and phosphatidylethanolamine (PE) were increased. Given the strong hydrophobicity of phenanthrene, exposure to this compound can affect bacterial membrane structure and membrane protein function. Bacteria can generally maintain membrane structural and functional stability by modulating membrane lipid composition [31]. Therefore, the increased levels of PC and PE may reflect enhanced membrane lipid biosynthesis and membrane remodeling in GHP1, potentially contributing to its tolerance to phenanthrene stress [32].

Taken together, the differential metabolite profiles, correlation analysis, and KEGG pathway enrichment results suggest that phenanthrene degradation by GHP1 may involve two potential metabolic routes: the phthalate pathway and the salicylate pathway. This hypothesis is broadly consistent with previously reported microbial degradation patterns of PAHs [33,34]. However, because the present study was primarily based on metabolomic data, the specific enzyme systems and key functional genes involved in these pathways cannot yet be conclusively determined. Further studies integrating transcriptomic analysis, detection of key degradation genes, and enzyme activity assays are therefore needed to validate the molecular mechanisms underlying phenanthrene degradation by GHP1.

### 3.2. Responses of Lettuce Growth and Physiology to Phenanthrene Stress

Phenanthrene, a representative polycyclic aromatic hydrocarbon (PAH), is highly hydrophobic and potentially toxic to plants. It can impair root absorption, induce the accumulation of reactive oxygen species (ROS), and disrupt chloroplast structure, thereby inhibiting plant growth, reducing photosynthetic efficiency, and promoting the accumulation of pollutants in plant tissues [35,36]. In the present study, under 50 mg·kg^−1^ phenanthrene stress, lettuce plants inoculated with strain GHP1 showed significantly greater root length, root fresh weight, root dry weight, and aboveground growth than those in the uninoculated control, accompanied by markedly higher chlorophyll a and chlorophyll b contents (Figure 6). These results indicate that GHP1 inoculation effectively alleviated the adverse effects of phenanthrene stress on lettuce growth and photosynthetic physiology.

After entering plant roots, phenanthrene can be transported from the root surface to aboveground tissues through root adsorption, active uptake, and transpiration, thereby exerting toxic effects within plant tissues [37,38]. In the present study, inoculation with GHP1 markedly reduced the residual phenanthrene levels in both lettuce roots and leaves, indicating that GHP1 may reduce plant exposure to phenanthrene under contaminated conditions. Previous studies have shown that the vicinity of plant roots is generally a hotspot for PAH degradation, which is closely associated with the activities of rhizospheric and endophytic microorganisms [39]. The ability of PAH-degrading bacteria to attach to hydrophobic surfaces, produce biosurfactants, and form biofilms can enhance the bioavailability and degradation efficiency of PAHs [40]. It has been reported that GHP1 possesses a certain capacity for biofilm formation [16]. Therefore, GHP1 likely enhances its contact with and degradation of phenanthrene through colonization of the root surface. However, this inference remains speculative, as 16S rRNA sequencing cannot distinguish the inoculated strain from indigenous *Sphingobium* species. Endophytic bacteria capable of degrading PAHs can degrade pollutants that enter plant tissues or accumulate near the root surface, thereby reducing pollutant uptake and accumulation by plants [41,42]. Thus, GHP1 may alleviate phenanthrene toxicity to lettuce by directly degrading phenanthrene and reducing its bioavailable concentration around the roots and within plant tissues, consequently decreasing phenanthrene uptake and subsequent translocation within the plant.

### 3.3. Effects of GHP1 Inoculation on the Root Endophytic Bacterial Community of Lettuce Under Phenanthrene Stress

Following GHP1 inoculation, the composition of the endophytic bacterial community in lettuce roots changed markedly. Alpha diversity analysis showed that the Sobs and Chao1 indices increased, whereas the Simpson index decreased in the P group compared with the control (Table 3), suggesting that GHP1 introduction increased the species richness of the root endophytic bacterial community and altered the distribution pattern of dominant taxa. Similar phenomena have been reported in studies of microorganisms involved in pollution remediation. The introduction of exogenous functional bacteria into plant–microbe systems can not only directly contribute to pollutant degradation but may also alter resource competition, niche structure, and the root microenvironment, thereby promoting the enrichment of environmentally adapted microbial taxa [32].

At the community composition level, GHP1 inoculation promoted the enrichment of bacterial taxa potentially associated with pollutant adaptation or degradation. At the phylum level, Pseudomonadota was the dominant phylum in the control, whereas the relative abundances of Actinobacteriota and Firmicutes increased following GHP1 inoculation (Figure 7a). Previous studies have indicated that Actinobacteriota and Pseudomonadota are important functional groups in environmental pollution remediation systems and contain numerous members capable of degrading PAHs, aromatic compounds, and other organic pollutants [43]. At the genus level, the relative abundances of *Nesterenkonia*, *Halomonas*, and *Dietzia* increased in the GHP1-treated group (Figure 7b). Members of the genus *Dietzia* have been reported to possess the potential to degrade hydrocarbon pollutants, whereas some members of *Nesterenkonia* and *Halomonas* exhibit strong environmental adaptability and may contribute to maintaining microbial community stability under pollutant stress [44,45,46]. Therefore, the enrichment of these taxa may reflect changes in the root endophytic microenvironment following GHP1 inoculation and the subsequent promotion of microbial groups with potential pollutant-adaptation or degradation capabilities.

Venn analysis showed that the control and P groups shared 21 core OTUs (Figure 8), indicating the presence of relatively stable core microbial taxa within lettuce roots. Meanwhile, the P group contained 108 unique OTUs (Figure 8), suggesting that GHP1 inoculation altered the existing microbial niche and may have promoted the recruitment or enrichment of additional bacterial taxa. LEfSe analysis further showed that the control was mainly characterized by the enrichment of indigenous taxa such as *Sphingobium* and Enterobacteriaceae, whereas *Dietzia*, *Nesterenkonia*, *Burkholderia*, and *Halomonas* showed an enrichment trend in the P group (Figure 9). These findings suggest that GHP1 may not only function as a pollutant-degrading bacterium directly involved in phenanthrene removal but may also reshape the internal root microenvironment and promote the reorganization of specific microbial communities, thereby potentially enhancing the overall adaptation of the plant–microbe system to phenanthrene stress.

Taken together, the remediation effect of GHP1 on lettuce under phenanthrene contamination may involve two major aspects. First, GHP1 can utilize phenanthrene as a carbon source and reduce phenanthrene levels in the surrounding environment and plant tissues through aromatic compound degradation pathways, thereby alleviating phenanthrene-induced phytotoxicity. Second, GHP1 inoculation can alter the structure of the root endophytic bacterial community and increase the relative abundance of microbial taxa potentially associated with pollutant adaptation or degradation. The combined effects of direct pollutant degradation and microbial community restructuring may contribute to enhancing the overall tolerance and remediation capacity of the plant–microbe system under phenanthrene stress.

The ecological safety and environmental persistence of GHP1 also require further evaluation. GHP1 inoculation altered the relative abundances of several bacterial phyla in lettuce roots, but *Sphingobium* was not significantly enriched in the P treatment. The introduction of exogenous functional bacterial strains may potentially cause adverse disturbances to the indigenous endophytic microbial community, potentially resulting in the loss of certain key native taxa and disrupting the existing balance of the endophytic microbiota. The long-term survival and persistence of the inoculated strain in the environment remain uncertain. In addition, the release of the strain into the natural environment may pose potential ecological risks, as it may affect non-target plants and, if it persists over the long term, continuously alter the composition of soil microbial communities. In addition, the physiological antioxidant responses of plants were not determined in the present study, which should be further explored in future investigations. Future studies using strain-specific qPCR or genome-based tracking, together with long-term soil and field experiments involving different plant species, are needed to assess GHP1 colonization, persistence, and potential effects on indigenous microbial communities. Overall, the present study provides preliminary evidence for the application potential of GHP1 in phenanthrene-contaminated plant systems, while its long-term ecological effects remain to be clarified.

## 4. Materials and Methods

### 4.1. Reagents, Culture Media and Equipment

Phenanthrene (purity ≥ 98%) was purchased from Sigma-Aldrich (St. Louis, MO, USA), while methanol and n-hexane were purchased from Fisher Scientific (Pittsburgh, PA, USA). Other reagents used in this study included acetone, 30% NaOH solution, calcium carbonate and quartz sand, boric acid solution, methyl red–bromocresol green mixed indicator, 0.02020202 mol·L^−1^ sulfuric acid standard solution, sodium hypochlorite solution containing 2.5% available chlorine, and 75% ethanol. All organic solvents were of HPLC grade, and all inorganic chemical reagents were of analytical grade or higher. Solutions requiring sterilization were sterilized by filtration through 0.22 μm membrane filters. DNA was extracted using the FastPure Plant DNA Isolation Mini Kit (DC104-01, Vazyme, Nanjing, China).

Murashige and Skoog (MS) basal medium was prepared by dissolving 9.4 g·L^−1^ MS powder and 10 g·L^−1^ sucrose in deionized water, adjusting the pH to 5.80, and adding 10 g·L^−1^ agar. The medium was sterilized by autoclaving at 115 °C for 30 min.

Luria–Bertani (LB) medium contained 10 g tryptone, 5 g yeast extract, and 10 g NaCl per liter of distilled water, with the pH adjusted to 7.0. The medium was sterilized at 121 °C for 15 min. For solid LB medium, 15 g·L^−1^ agar was additionally added [47].

Mineral salt medium (MSM) contained 0.02 g CaCl_2_, 0.2 g MgSO_4_, 0.5 g NaNO_3_, 0.5 g (NH_4_)_2_SO_4_, 1 g KH_2_PO_4_, 1 g NaH_2_PO_4_·H_2_O, and 15 g agar per liter of distilled water, with the pH adjusted to 6.5. The medium was sterilized at 121 °C for 15 min [48]. For solid MSM, 15 g·L^−1^ agar was additionally added.

Equipment: Super-clean workbench (SW-CJ-2D, Suzhou Purification Equipment Co., Ltd., Suzhou, China); Constant-temperature shaker (THZ-98AB, Shanghai Yiheng Scientific Instrument Co., Ltd., Shanghai, China); high-speed refrigerated centrifuge (HC-3016B, Anhui Zhongke Zhongjia Scientific Instrument Co., Ltd., Hefei, China); vortex mixer (Vortex-Genie Pulse, Scientific Industries, Bohemia, NY, USA); autoclave (YXQ-LB, Shanghai Boxun Industrial Co., Ltd., Shanghai, China); UV-visible spectrophotometer (C-7202S, Piken, Shanghai, China); high-performance liquid chromatography (HPLC, EClassical 3200, Elite-HPLC, Suzhou, China); Nitrogen-blow concentrator (WT-12A, Miou, Hangzhou, China); ultrasonic cleaner (XM-P221H, Xiaomei, Kunshan, China); constant-temperature incubator (DH-420A, Beijing Zhongxing Weiye Instrument Co., Ltd., Beijing, China); pH meter (PHSJ-4F, Shanghai Inesa Scientific Instrument Co., Ltd., Shanghai, China); analytical balance (FA2004, Shanghai Suny Hengping Scientific Instrument Co., Ltd., Shanghai, China).

### 4.2. Bacterial Strain

The bacterial strain GHP1 used in this study was a phenanthrene-degrading strain previously isolated, screened, and preserved in the laboratory. GHP1 is a Gram-negative, rod-shaped, non-spore-forming bacterium with a single polar flagellum. Based on taxonomic identification, the strain was identified as *Sphingobium abikonense*, with GenBank accession number MW345956.

### 4.3. Phenanthrene Degradation by Strain GHP1

The phenanthrene degradation characteristics of strain GHP1 were determined according to the method described by Sun et al. [49], using phenanthrene as the sole carbon source in liquid degradation assays. The strain was first activated in LB medium at 28 °C and 130 rpm for 36 h. Cells were subsequently harvested by centrifugation at 8000 rpm at 4 °C, washed twice with MSM, and resuspended. The cell suspension was adjusted to an OD_600_ of 1.0, corresponding to approximately 10^12^ CFU [50].

A phenanthrene stock solution in acetone was added to sterile Erlenmeyer flasks, and the acetone was allowed to evaporate completely. MSM degradation medium was then prepared at a final phenanthrene concentration of 50 mg·L^−1^. GHP1 was inoculated at a ratio of 1 mL bacterial suspension to 9 mL degradation medium and incubated at 28 °C and 130 rpm in the dark. Samples were collected at 0, 1, 3, and 5 d. The OD_600_ was measured to characterize bacterial growth. Samples were extracted with an equal volume of methanol, sonicated for 30 min, and filtered through 0.22 μm PTFE membrane filters before determination of residual phenanthrene by HPLC.

HPLC analysis was performed using a Symmetry C18 column (Waters, Milford, MA, USA) with methanol/water (90:10, *v*/*v*) as the mobile phase at a flow rate of 1 mL·min^−1^. The column temperature was maintained at 30 °C, the injection volume was 50 μL, and the detection wavelength was 254 nm. The retention time of phenanthrene was 7.2 min, and the method recovery was 93.4 ± 0.11%. A sterile phenanthrene–MSM system without bacterial inoculation was used as the abiotic control. Three independent replicates were included for each treatment.

The degradation rate constant (*k*) and half-life (*t*_1_/_2_) were calculated using a first-order kinetic equation: *C* = *C*_0_ × e^−^*^kt^*. Where *C*_0_ and *C* represent the initial and residual phenanthrene concentrations, respectively. The half-life was calculated as: t_1_/_2_ = ln2/*k*. The phenanthrene residual rate (*RR*) was calculated as: *RR* = (*C*_0_ − *C*)/*C*_0_, according to Gu et al. [51].

### 4.4. Untargeted Metabolomic Analysis

#### 4.4.1. Sample Preparation and Analytical Procedures

Samples were collected on days 0, 1, and 3 according to the experimental procedures and treatment groups described in Section 4.3. After collection, samples were immediately transferred to centrifuge tubes and stored at −20 °C until further processing. The samples were subsequently submitted to Meiji Biological Technology Co., Ltd. (Shanghai, China) for untargeted metabolomic analysis.

For metabolite extraction, a 96-well protein precipitation plate was assembled with a collection plate and placed in a Starlid™ automated workstation (Bioyong Technologies, Shanghai, China). Then, 400 μL of extraction solvent containing internal standards and pre-cooled to −40 °C (methanol/acetonitrile, 1:1, *v*/*v*) was transferred to the protein precipitation plate, followed by the addition of 100 μL of each sample. The plate was shaken at 750 rpm for 5 min and allowed to stand for 5 min. After filtration, the filtrates were collected. Equal volumes of filtrates from all samples were pooled to prepare quality control (QC) samples for evaluating instrument stability and analytical performance [21].

Chromatographic separation was performed using a Vanquish ultra-high-performance liquid chromatography (UHPLC) system (Thermo Fisher Scientific, Waltham, MA, USA). Polar metabolites were separated using a Waters ACQUITY UPLC BEH Amide column (2.1 mm × 50 mm, 1.7 μm; Waters, Milford, MA, USA) [48]. Mobile phase A consisted of water containing 25 mmol·L^−1^ ammonium acetate and 25 mmol·L^−1^ ammonia, while mobile phase B was acetonitrile. The autosampler temperature was maintained at 4 °C, and the injection volume was 2 μL.

Mass spectrometric analysis was performed using an Orbitrap Exploris 120 mass spectrometer (Thermo Fisher Scientific, Waltham, MA, USA). Full-scan and MS/MS data were acquired using Xcalibur 4.4 software (Thermo Fisher Scientific, Waltham, MA, USA). The main MS parameters were as follows: sheath gas flow rate, 50 Arb; auxiliary gas flow rate, 15 Arb; capillary temperature, 320 °C; MS1 resolution, 60,000; MS2 resolution, 15,000; stepped normalized collision energy (SNCE), 20/30/40; spray voltage, 3.8 kV in positive ion mode and −3.4 kV in negative ion mode.

#### 4.4.2. Data Processing and Metabolite Annotation

Individual peaks were initially filtered to remove background noise and low-quality signals. Subsequently, abnormal peaks and outliers were filtered based on the relative standard deviation (RSD), i.e., coefficient of variation (CV). Peak-area data were retained when the proportion of missing values within a single group was ≤50% or when the proportion of missing values across all groups was ≤50%. Missing values in the raw dataset were imputed using half of the minimum detected value, and the data were normalized based on internal standards [52].

Metabolomic data were analyzed separately in negative ion mode (NEG) and positive ion mode (POS). Differential metabolites were identified using the following criteria: Student’s *t*-test *p* < 0.05 and a variable importance in projection (VIP) score >1 from the first principal component of the OPLS-DA model. The screening results were visualized using volcano plots.

Hierarchical clustering analysis of differential metabolites was performed based on their quantitative values in each pairwise group comparison. Euclidean distance matrices were first calculated, followed by clustering using the complete-linkage method. The results were presented as heatmaps. Correlation analysis of differential metabolites was performed using Pearson’s correlation analysis based on their quantitative values, and the results were visualized as correlation heatmaps.

Functional annotation and pathway analysis of differential metabolites were conducted based on the Kyoto Encyclopedia of Genes and Genomes (KEGG) database. Differential metabolites were matched to KEGG metabolic pathways to investigate the biological metabolic processes in which they may participate. In pathway maps, upregulated metabolites were highlighted in bright red, whereas downregulated metabolites were highlighted in bright blue.

### 4.5. Lettuce Seedling Cultivation

Strain GHP1 was cultured in LB liquid medium at 28 °C and 130 rpm for 48 h. Cells were harvested by centrifugation and washed with sterile water to remove residual LB medium. The cells were subsequently resuspended in sterile water, and the bacterial suspension was adjusted to an OD_600_ of 0.6 for subsequent inoculation.

Lettuce seed surface sterilization was performed according to Liu [53]. Briefly, lettuce seeds were immersed in 70% ethanol for 30 s, followed by sterilization in sodium hypochlorite solution containing 2.5% available chlorine for 20 min. The seeds were then rinsed 3–5 times with sterile distilled water to obtain surface-sterilized seeds.

Two treatment groups were established: a sterile water treatment (control) and a GHP1 bacterial suspension treatment (P). Surface-sterilized seeds were immersed in sterile water or the GHP1 suspension for 8 h. After treatment, the seeds were aseptically placed onto MS medium using sterile tweezers and toothpicks, and the Petri dishes were sealed with sealing film. The plates were first maintained at 4 °C in the dark for 24 h and then transferred to a light growth chamber and cultured for 42 d at 28 °C, a light intensity of 1500–2000 lux, a relative humidity of 60%, and a photoperiod of 12 h·d^−1^. At the end of the cultivation period, the effects of the different treatments on lettuce growth and physiological parameters were evaluated.

#### 4.5.1. Growth and Physiological Responses of Lettuce to Phenanthrene Stress

After 42 d of cultivation, the fresh weight, dry weight, number of root branches, leaf length, leaf width, root length, and chlorophyll content of lettuce plants were determined for each treatment. Root length, shoot length, and leaf width were measured using a flexible ruler. Fresh weight was determined immediately after harvesting, whereas dry weight was determined after samples were killed at 105 °C for 30 min and subsequently dried at 55 °C to constant weight. Biological replicates were established for each treatment, and the mean value of each parameter was calculated.

Chlorophyll content was determined according to the method described by Ngcobo et al. [54]. Briefly, 0.5 g of fresh lettuce leaves was weighed and ground thoroughly in a mortar with 5 mL of pure acetone, a small amount of calcium carbonate, and quartz sand. Subsequently, 5 mL of 80% acetone was added. The suspension was transferred to a centrifuge tube and brought to a final volume of 20 mL with 80% acetone. Using 80% acetone as the blank, the absorbance of the extract was measured at 663 and 645 nm.

The concentrations of chlorophyll a, chlorophyll b, and total chlorophyll were calculated as follows: *C*_a_ = 12.71*A*_663_ − 2.59*A*_645_; *C*_b_ = 22.88*A*_645_ − 4.67*A*_663_; *C*_t_ = *C*_a_ + *C*_b_ = 8.04*A*_663_ + 20.29*A*_645_. The chlorophyll content of the samples was calculated as: Chlorophyll content (mg·g^−1^ FW) = *C* × *V* × (m × 1000)^−1^, where *A*_663_ and *A*_645_ represent the absorbance of the sample at 663 and 645 nm, respectively; C_a_ represents the concentration of chlorophyll a (mg·L^−1^); C_b_ represents the concentration of chlorophyll b (mg·L^−1^); C_t_ represents the total chlorophyll concentration (mg·L^−1^); C represents the corresponding chlorophyll concentration (mg·L^−1^); V represents the total volume of the extract (mL); and m represents the fresh weight of the sample (g).

#### 4.5.2. Determination of Residual Phenanthrene in Lettuce

Root and shoot tissues of lettuce plants were collected separately and subjected to freeze-drying. Precisely 0.1 g of each dried sample was weighed and thoroughly ground. The samples were extracted by ultrasonication for 30 min using an equal-volume solvent mixture as the extraction solvent. After extraction, the supernatant was separated and collected, and the residue was supplemented with the same proportion of extraction solvent for repeated ultrasonic extraction. The extraction procedure was repeated three times in total.

The three extracts were combined and sequentially dehydrated with anhydrous sodium sulfate and purified using a silica gel solid-phase column. The silica gel column was subsequently eluted with 10 mL of an equal-volume mixture of n-hexane and dichloromethane. The eluate was collected and combined with the previously purified extract in a nitrogen-blowdown tube. The combined extract was evaporated to dryness under a high-purity nitrogen stream, and the residue was redissolved in 2.00 mL methanol. The solution was vortexed until the residue was completely dissolved and then filtered through a 0.22 μm polytetrafluoroethylene (PTFE) membrane filter. A 1.0 mL aliquot of the filtrate was transferred to an HPLC vial, and the residual phenanthrene concentration was quantitatively determined by HPLC [55].

#### 4.5.3. Response of the Lettuce Root Endophytic Bacterial Community to Phenanthrene Stress

Genomic DNA was extracted strictly according to the instructions provided with the corresponding DNA extraction kit. The integrity of genomic DNA was assessed by 1% agarose gel electrophoresis, and DNA concentration and purity were determined using a NanoDrop One spectrophotometer (Thermo Fisher Scientific, Wilmington, DE, USA). Qualified genomic DNA was used as the template for PCR amplification. Barcode-containing specific primers were selected according to the target sequencing region, and PCR amplification was performed using the Premix Taq (TaKaRa, Beijing, China) system.

The PCR products were examined by agarose gel electrophoresis, and their relative quantities were determined using GeneTools Analysis Software (Version 4.03.05.0, SynGene, Cambridge, UK). Based on the principle of equal-mass pooling, the required volume of PCR product from each sample was calculated, and equal amounts of amplification products from all samples were pooled. The pooled PCR products were subsequently recovered from agarose gels using the E.Z.N.A.^®^ Gel Extraction Kit (Omega Bio-Tek, Norcross, GA, USA) and eluted with TE buffer to obtain the target DNA fragments.

Libraries were constructed according to the standard protocol of the NEBNext^®^ Ultra™ DNA Library Prep Kit for Illumina^®^ (New England Biolabs, Ipswich, MA, USA), followed by paired-end sequencing on an Illumina HiSeq/MiSeq high-throughput sequencing platform. Raw image data generated by the sequencing platform (Illumina, San Diego, CA, USA) were converted into raw sequencing reads through base calling and stored in FASTQ format, which contains the sequencing reads and their corresponding quality scores.

The paired-end raw reads were subjected to quality filtering and preprocessing. First, fastp was used for sliding-window quality trimming of the paired-end raw reads. Primer sequences were removed using cutadapt based on the primer information at both ends of the sequences, generating paired-end clean reads. The clean reads were then merged using usearch -fastq_mergepairs based on the overlap between paired-end sequences, and sequences that did not meet the merging criteria were discarded to obtain raw tags. The raw tags were subsequently subjected to sliding-window quality filtering using fastp with the parameters W = 4 and M = 20 to remove low-quality sequences, generating high-quality clean tags.

To characterize the taxonomic composition and diversity of the microbial communities, clean tags from all samples were clustered into operational taxonomic units (OTUs), and chimeric sequences were removed simultaneously. Representative sequences from each OTU were aligned against the reference database for taxonomic annotation, and the corresponding taxonomic information was obtained. The resulting data were subsequently analyzed using the relevant cloud-based platform.

## 5. Conclusions

By integrating liquid-phase phenanthrene degradation experiments, untargeted metabolomics, lettuce cultivation assays, and analysis of root endophytic bacterial communities, this study demonstrated that *Sphingobium abikonense* GHP1 can rapidly degrade phenanthrene under phenanthrene stress, potentially through the phthalate and salicylate degradation pathways, accompanied by coordinated metabolic responses. Specifically, the accumulation of 1-hydroxy-2-naphthoic acid, salicylic acid, and hydroxybenzoic acid derivatives, together with the activation of 2-oxoacid metabolism and aromatic compound degradation pathways, suggested that GHP1 may adapt to phenanthrene stress by promoting pollutant degradation, central carbon metabolism, redox regulation, and membrane lipid metabolism.

In the lettuce cultivation system, GHP1 inoculation promoted plant growth, increased root biomass and chlorophyll contents, and reduced phenanthrene residues in plant tissues. Meanwhile, GHP1 inoculation altered the composition of the root endophytic bacterial community and promoted the enrichment of certain environmentally adapted bacterial taxa.

Overall, GHP1 may enhance the remediation capacity of the plant–microbe system under phenanthrene contamination through multiple complementary mechanisms, including direct phenanthrene degradation, regulation of bacterial metabolic responses, and modulation of the root endophytic microbial community. These findings provide a theoretical basis and a potential microbial resource for the development of plant–microbe-based bioremediation strategies for phenanthrene-contaminated environments.

## Figures and Tables

**Table 1:** First-order degradation kinetic model.

	*k*	*t*_1/2_ (d)	*R* ^2^
Control	0.0209	33.23	0.141
P	1.125	0.616	0.99

**Table 2:** Phenanthrene concentrations in the root and shoot of lettuce under the control and P treatments.

	Phenanthrene Content (mg·kg^−1^)		*p* Value
	Control	P	
Root	118.83 ± 5.64	33.23	0.0013
Shoot	46.79 ± 5.19	52.61 ± 11.45	0.66

**Table 3:** Alpha Diversity of Endophytic Bacterial Communities in Lettuce Roots.

	Sobs	Chao1	ACE	Simpson
Control	49.67 ± 16.62 ab	49.67 ± 11.67 ab	49.67 ± 11.67 ab	0.1326 ± 0.0741 a
P	55.33 ± 10.11 a	55.33 ± 10.12 a	55.33 ± 10.12 a	0.0748 ± 0.0197 a

Note: Values followed by the same letter are not significantly different; different letters indicate significant differences; *p* < 0.05.

## Data Availability

The data presented in this study are available within the article. Additional data are available from the corresponding author upon reasonable request.

## Supplementary Materials

The following supporting information can be downloaded at: https://www.mdpi.com/article/10.3390/plants15203136/s1, Figure S1: Volcano plot comparison between P group and control. NEG mode at day 0 (a) and POS mode (b); POS mode at day 1 (c); NEG mode at day 3 (d) and POS mode (e).; Figure S2: Heatmap of differential metabolites in P vs. Control. NEG mode at day 0 (a) and POS mode (b); POS mode at day 1 (c); NEG mode at day 3 (d) and POS mode (e). Metabolites marked in red indicate the key substances mentioned in the text; Figure S3: Correlation analysis of differential metabolites in P vs. Control. NEG mode at day 0 (a) and POS mode (b); POS mode at day 1 (c); NEG mode at day 3 (d) and POS mode (e). Symbols marked with “×” indicate correlations with |r| < 0.6; Figure S4: KEGG enrichment plot of differential metabolites between P group and control. NEG mode at day 0 (a) and POS mode (b); POS mode at day 1 (c); NEG mode at day 3 (d) and POS mode (e). Metabolites marked in red indicate the key substances mentioned in the text.
