# Supplementary material for: Metabolic Regulation of Phenanthrene-Degrading Strain Sphingobium abikonense GHP1 in Response to Phenanthrene Stress and Its Effects on Endorhizosphere Microbiota of Lettuce
Source: Plants (Basel). 2026 Oct 14;15(20):3136. doi: 10.3390/plants15203136 (PMC13708759; doi:10.3390/plants15203136)
Supplement: Supplementary file 1 [file plants-15-03136-s001.zip › plants-4553647-supplementary.pdf]

Supporting Information

Metabolic Regulation of Phenanthrene-Degrading Strain *Sphingobium abikonense* GHP1 in Response to Phenanthrene Stress and Its Effects on Endorhizosphere Microbiota of Lettuce

Lihang Zhang, Zhangkai Wang, Yizhen Jia, Ling'en Liu, Yi Yang, Weizheng Ren and Haiping Gu *

College of Forestry, Henan Agricultural University, Zhengzhou 450046, China; zlihang06@163.com (L.Z.)
* Correspondence: hpg@henau.edu.cn

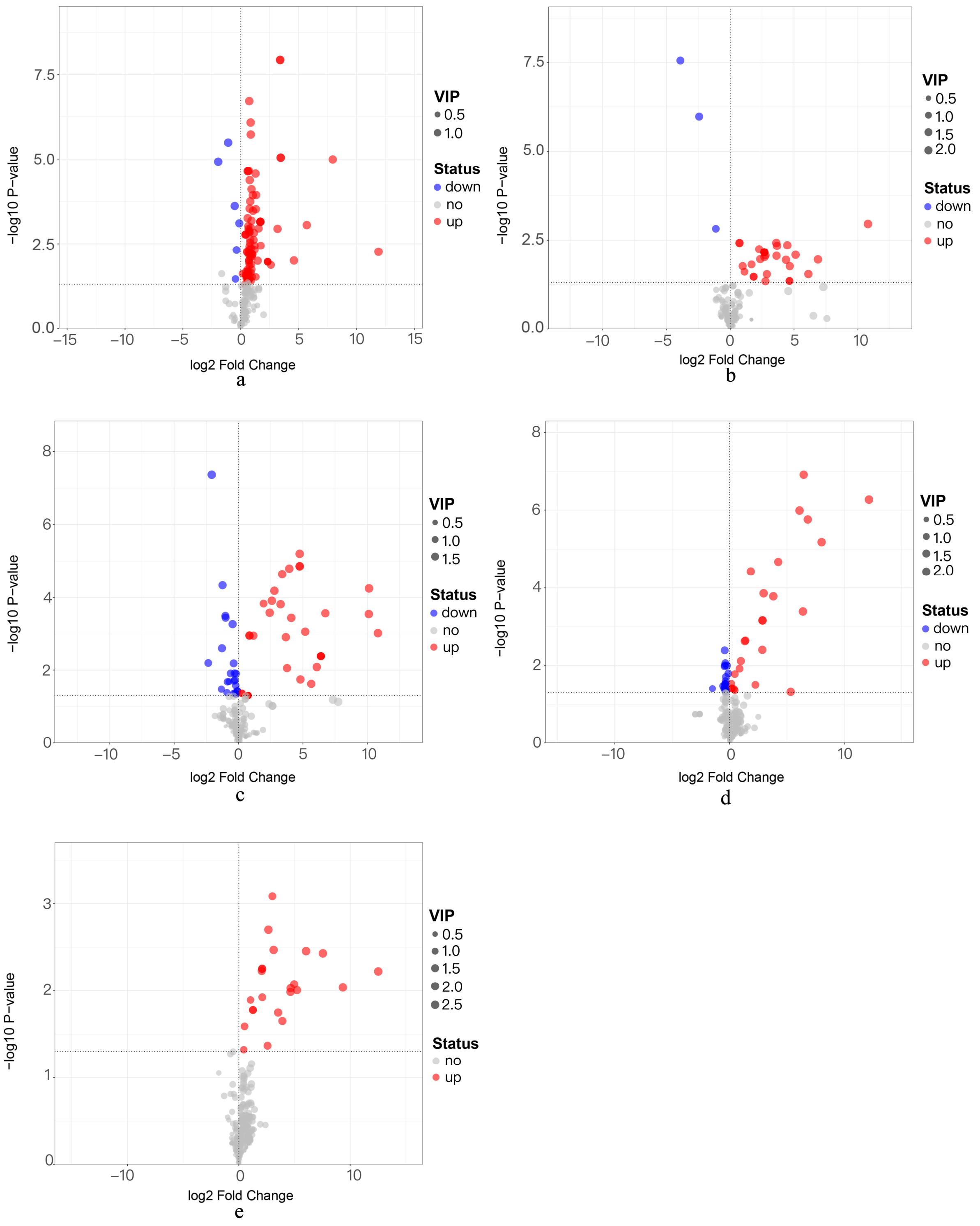

Figure S1. Volcano plot comparison between P group and control. NEG mode at day 0 (a) and POS mode (b); POS mode at day 1 (c); NEG mode at day 3 (d) and POS mode (e).

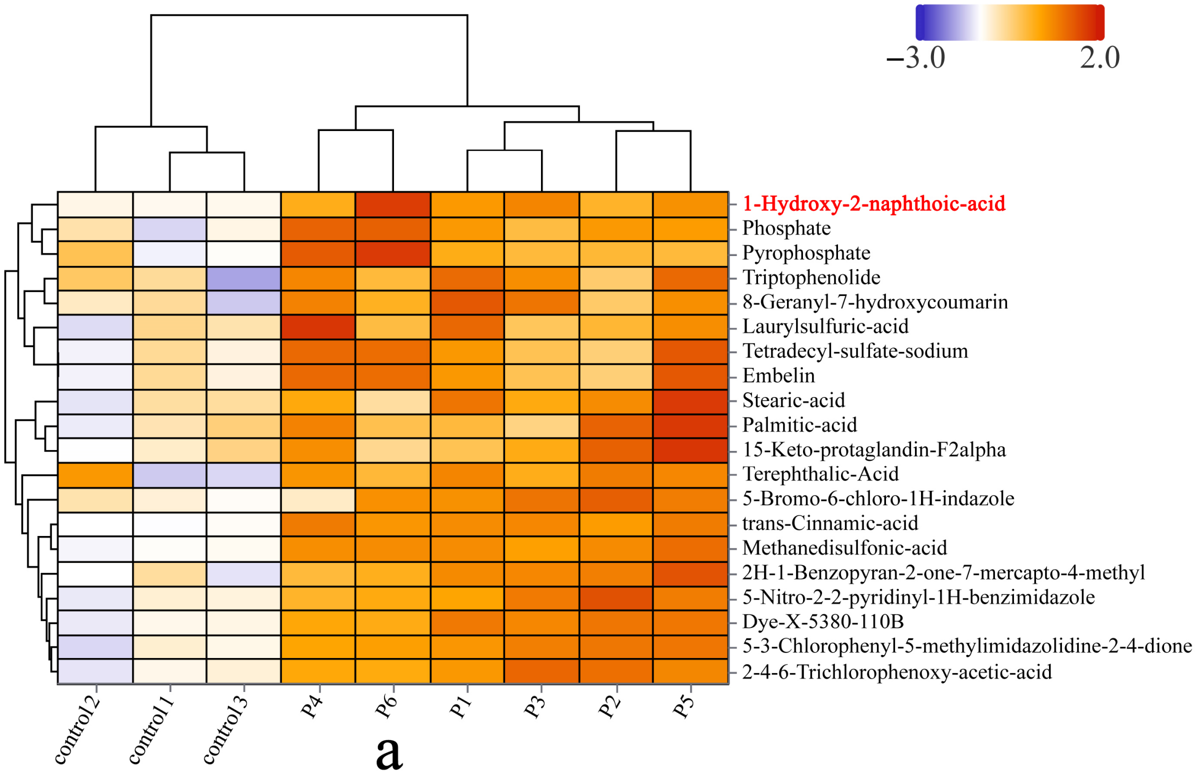
−3.0
2.0
1-Hydroxy-2-naphthoic-acid
Phosphate
Pyrophosphate
Triptophenolide
8-Geranyl-7-hydroxycoumarin
Laurylsulfuric-acid
Tetradecyl-sulfate-sodium
Embelin
Stearic-acid
Palmitic-acid
15-Keto-protaglandin-F2alpha
Terephthalic-Acid
5-Bromo-6-chloro-1H-indazole
trans-Cinnamic-acid
Methanedisulfonic-acid
2H-1-Benzopyran-2-one-7-mercapto-4-methyl
5-Nitro-2-2-pyridinyl-1H-benzimidazole
Dye-X-5380-110B
5-3-Chlorophenyl-5-methylimidazolidine-2-4-dione
2-4-6-Trichlorophenoxy-acetic-acid
control2
control1
control3
P4
P6
P1
P3
P2
P5

a

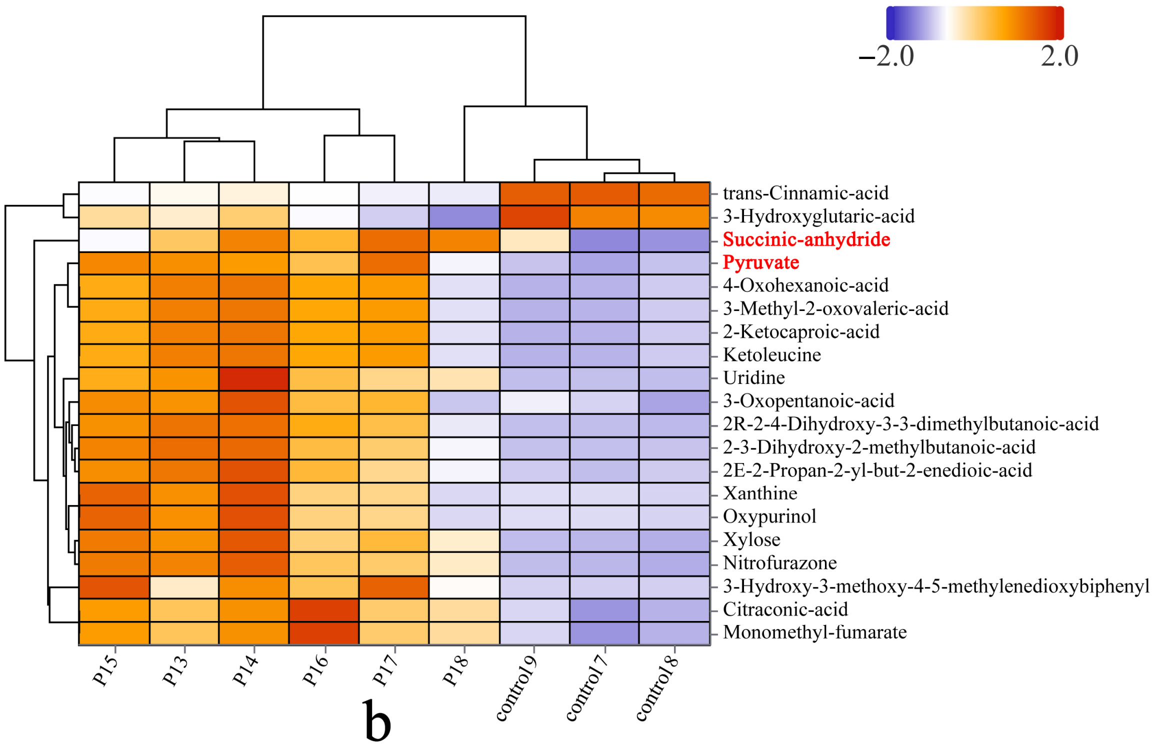
−2.0
2.0
trans-Cinnamic-acid
3-Hydroxyglutaric-acid
Succinic-anhydride
Pyruvate
4-Oxohexanoic-acid
3-Methyl-2-oxovaleric-acid
2-Ketocaproic-acid
Ketoleucine
Uridine
3-Oxopentanoic-acid
2R-2-4-Dihydroxy-3-3-dimethylbutanoic-acid
2-3-Dihydroxy-2-methylbutanoic-acid
2E-2-Propan-2-yl-but-2-enedioic-acid
Xanthine
Oxypurinol
Xylose
Nitrofurazone
3-Hydroxy-3-methoxy-4-5-methylenedioxybiphenyl
Citraconic-acid
Monomethyl-fumarate
P15
P13
P14
P16
P17
P18
control9
control7
control8

b

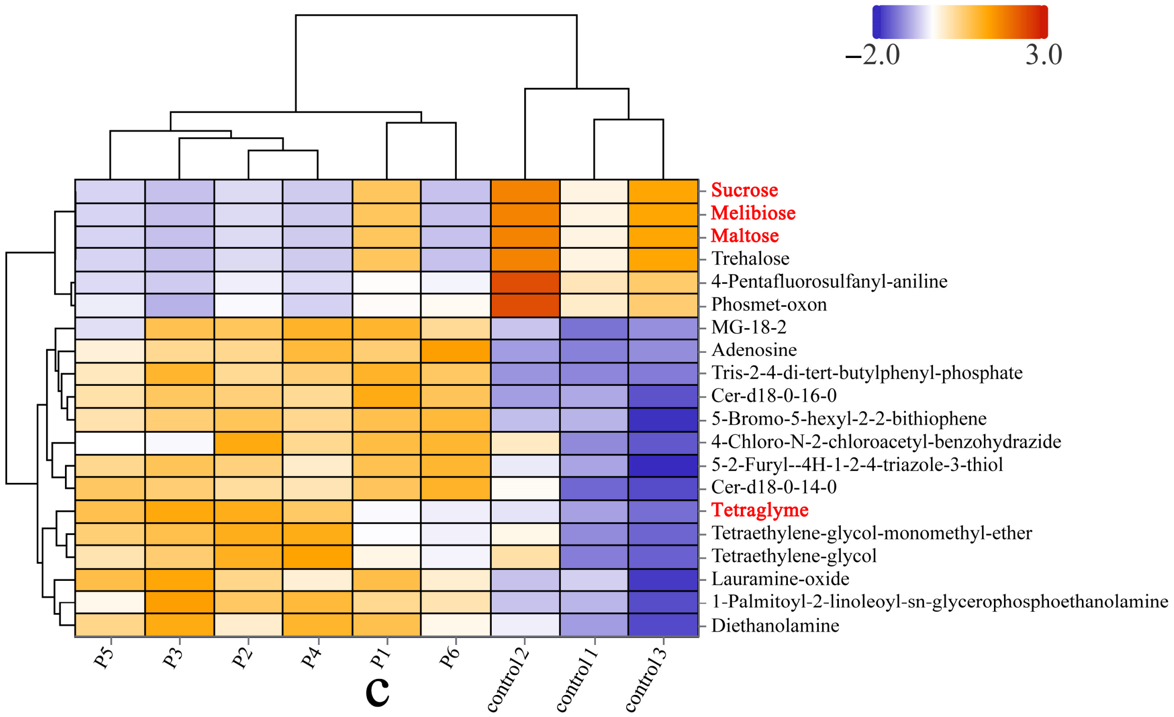

−2.0
3.0
Sucrose
Melibiose
Maltose
Trehalose
4-Pentafluorosulfanyl-aniline
Phosmet-oxon
MG-18-2
Adenosine
Tris-2-4-di-tert-butylphenyl-phosphate
Cer-d18-0-16-0
5-Bromo-5-hexyl-2-2-bithiophene
4-Chloro-N-2-chloroacetyl-benzohydrazide
5-2-Furyl--4H-1-2-4-triazole-3-thiol
Cer-d18-0-14-0
Tetraglyme
Tetraethylene-glycol-monomethyl-ether
Tetraethylene-glycol
Lauramine-oxide
1-Palmitoyl-2-linoleoyl-sn-glycerophosphoethanolamine
Diethanolamine
P5
P3
P2
P4
P1
P6
control 2
control 1
control 3

c

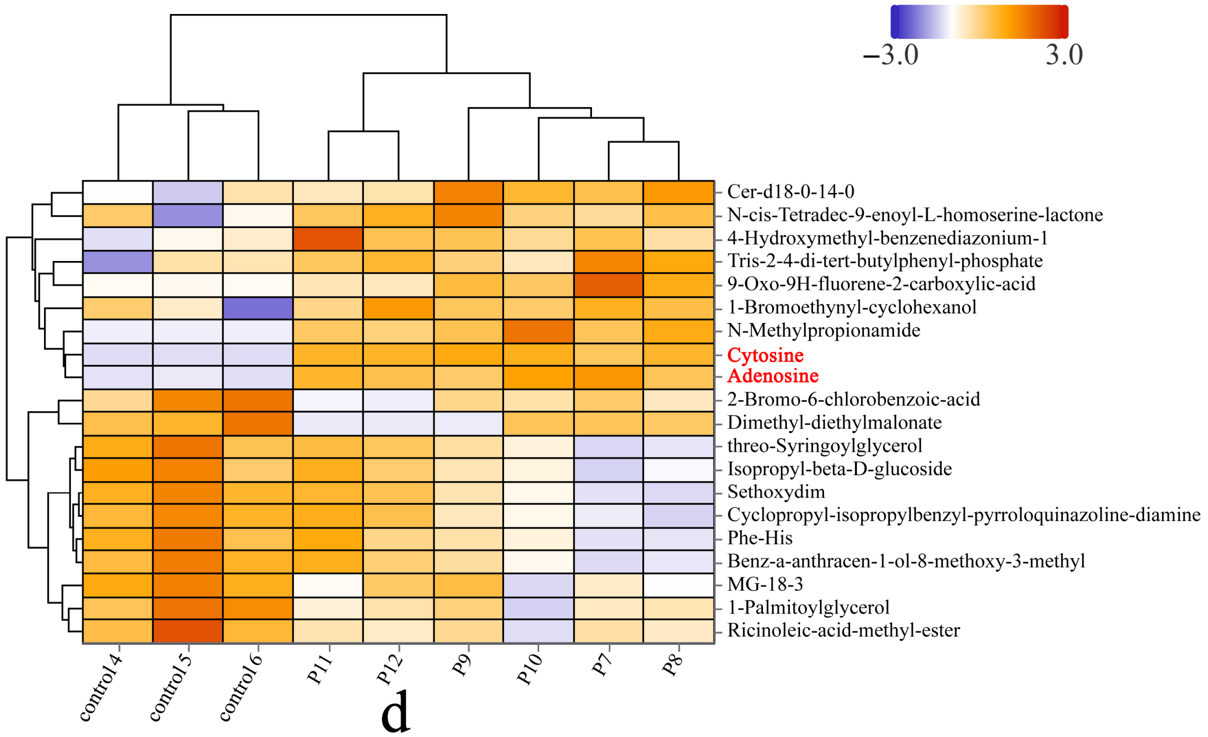

−3.0
3.0
Cer-d18-0-14-0
N-cis-Tetradec-9-enoyl-L-homoserine-lactone
4-Hydroxymethyl-benzenediazonium-1
Tris-2-4-di-tert-butylphenyl-phosphate
9-Oxo-9H-fluorene-2-carboxylic-acid
1-Bromoethynyl-cyclohexanol
N-Methylpropionamide
Cytosine
Adenosine
2-Bromo-6-chlorobenzoic-acid
Dimethyl-diethylmalonate
threo-Syringoylglycerol
Isopropyl-beta-D-glucoside
Sethoxydim
Cyclopropyl-isopropylbenzyl-pyrroloquinazoline-diamine
Phe-His
Benz-a-anthracen-1-ol-8-methoxy-3-methyl
MG-18-3
1-Palmitoylglycerol
Ricinoleic-acid-methyl-ester
control 4
control 5
control 6
P11
P12
P9
P10
P7
P8

d

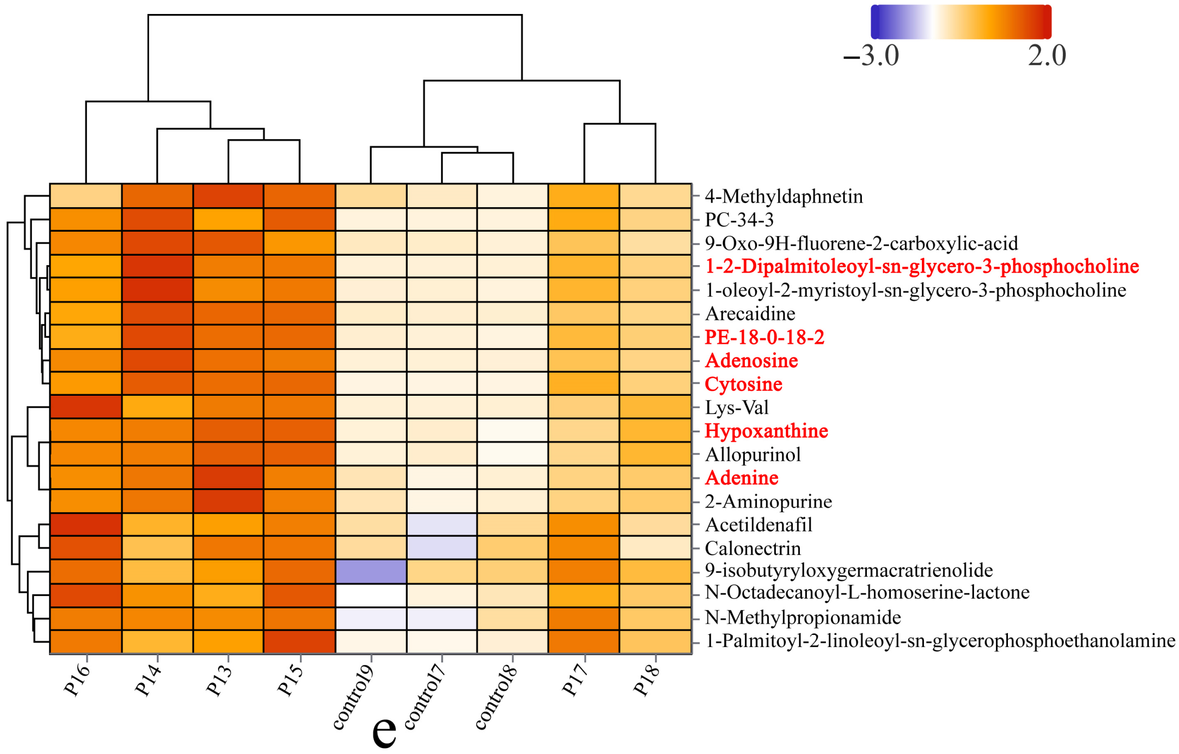

Figure S2. Heatmap of differential metabolites in P vs. Control . NEG mode at day 0 (a) and POS mode (b); POS mode at day 1 (c); NEG mode at day 3 (d) and POS mode (e). Metabolites marked in red indicate the key substances mentioned in the text.

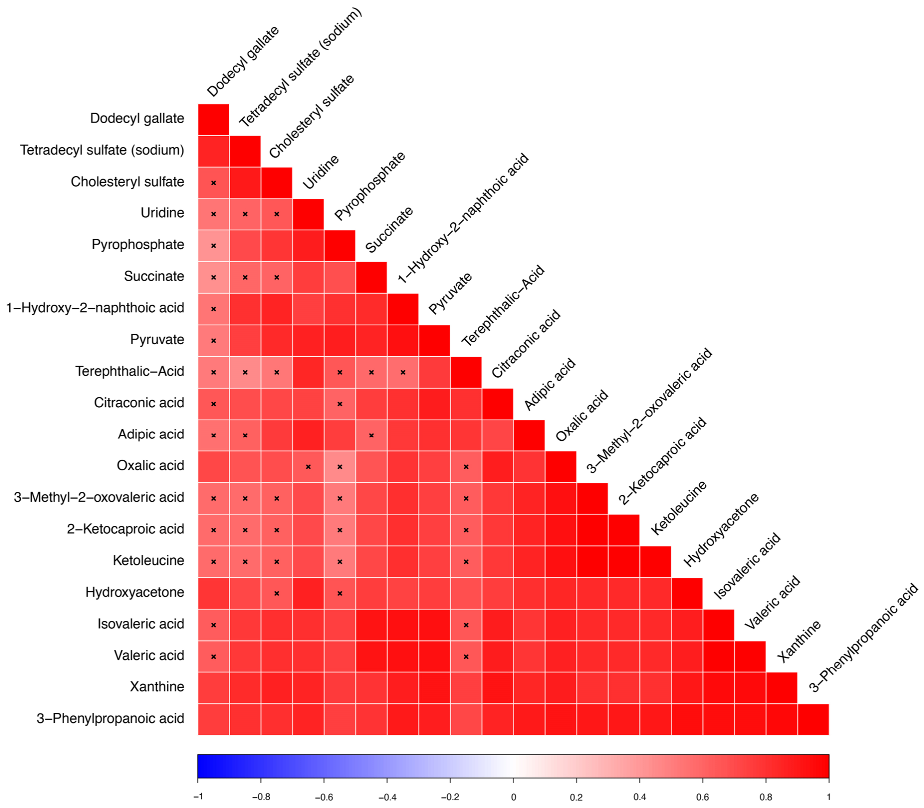

Dodecyl gallate
Tetradecyl sulfate (sodium)
Cholesteryl sulfate
Uridine
Pyrophosphate
Succinate
1−Hydroxy−2−naphthoic acid
Pyruvate
Terephthalic−Acid
Citraconic acid
Adipic acid
Oxalic acid
3−Methyl−2−oxovaleric acid
2−Ketocaproic acid
Ketoleucine
Hydroxyacetone
Isovaleric acid
Valeric acid
Xanthine
3−Phenylpropanoic acid
−1 −0.8 −0.6 −0.4 −0.2 0 0.2 0.4 0.6 0.8 1

a

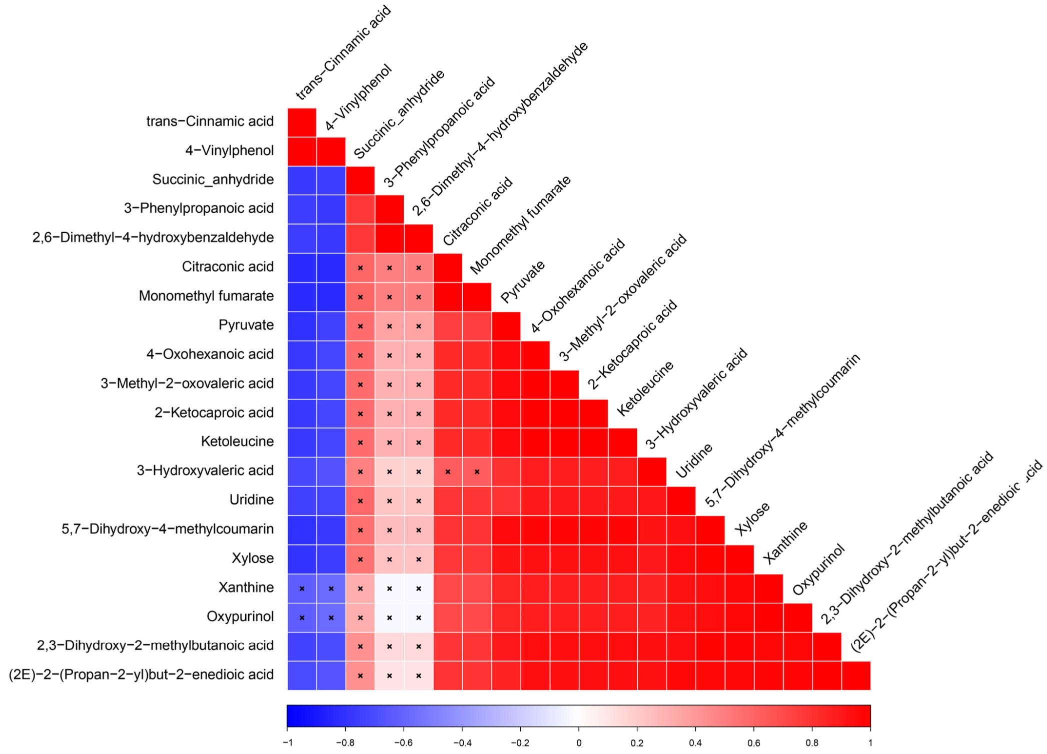

trans−Cinnamic acid
4−Vinylphenol
Succinic_anhydride
3−Phenylpropanoic acid
2,6−Dimethyl−4−hydroxybenzaldehyde
Citraconic acid
Monomethyl fumarate
Pyruvate
4−Oxohexanoic acid
3−Methyl−2−oxovaleric acid
2−Ketocaproic acid
Ketoleucine
3−Hydroxyvaleric acid
Uridine
5,7−Dihydroxy−4−methylcoumarin
Xylose
Xanthine
Oxypurinol
2,3−Dihydroxy−2−methylbutanoic acid
(2E)−2−(Propan−2−yl)but−2−enedioic acid
−1 −0.8 −0.6 −0.4 −0.2 0 0.2 0.4 0.6 0.8 1

b

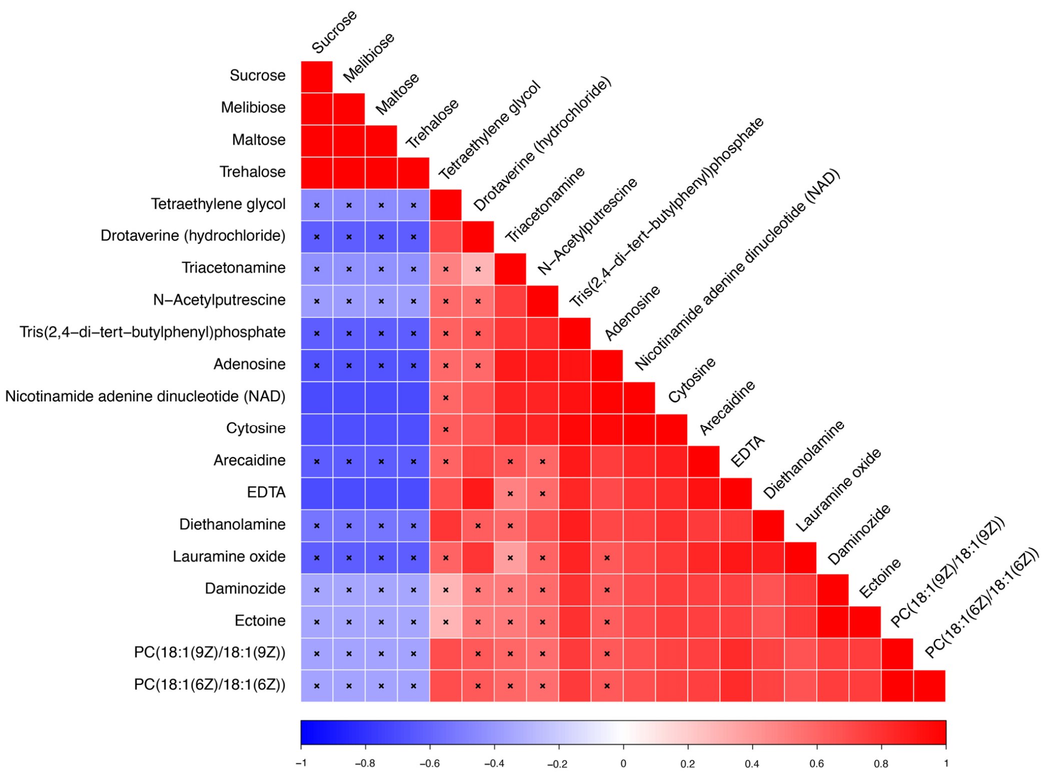
Sucrose
Melibiose
Maltose
Trehalose
Tetraethylene glycol
Drotaverine (hydrochloride)
Triacetonamine
N−Acetylputrescine
Tris(2,4−di−tert−butylphenyl)phosphate
Adenosine
Nicotinamide adenine dinucleotide (NAD)
Cytosine
Arecaidine
EDTA
Diethanolamine
Lauramine oxide
Daminozide
Ectoine
PC(18:1(9Z)/18:1(9Z))
PC(18:1(6Z)/18:1(6Z))
−1
−0.8
−0.6
−0.4
−0.2
0
0.2
0.4
0.6
0.8
1

c

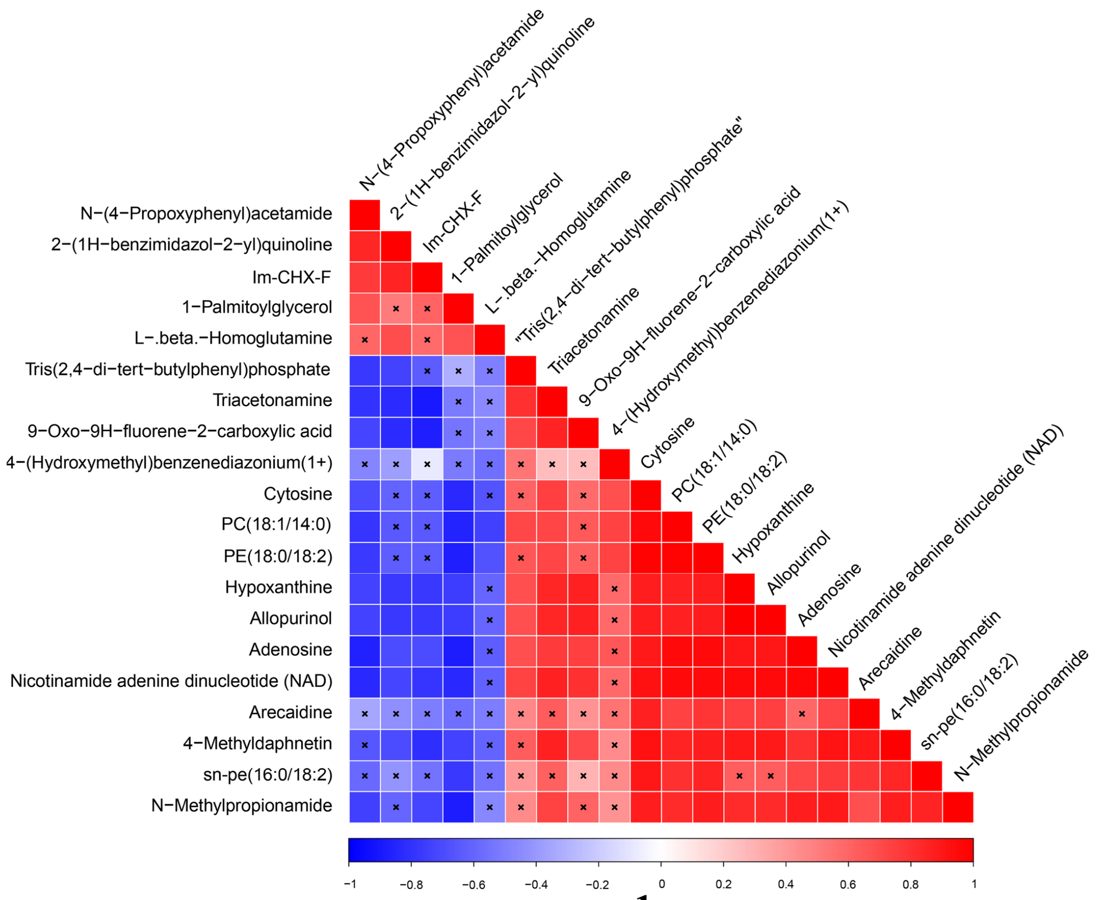
N−(4−Propoxyphenyl)acetamide
2−(1H−benzimidazol−2−yl)quinoline
Im-CHX-F
1−Palmitoylglycerol
L−.beta.−Homoglutamine
Tris(2,4−di−tert−butylphenyl)phosphate
Triacetonamine
9−Oxo−9H−fluorene−2−carboxylic acid
4−(Hydroxymethyl)benzenediazonium(1+)
Cytosine
PC(18:1/14:0)
PE(18:0/18:2)
Hypoxanthine
Allopurinol
Adenosine
Nicotinamide adenine dinucleotide (NAD)
Arecaidine
4−Methyldaphnetin
sn-pe(16:0/18:2)
N−Methylpropionamide
−1
−0.8
−0.6
−0.4
−0.2
0
0.2
0.4
0.6
0.8
1

d

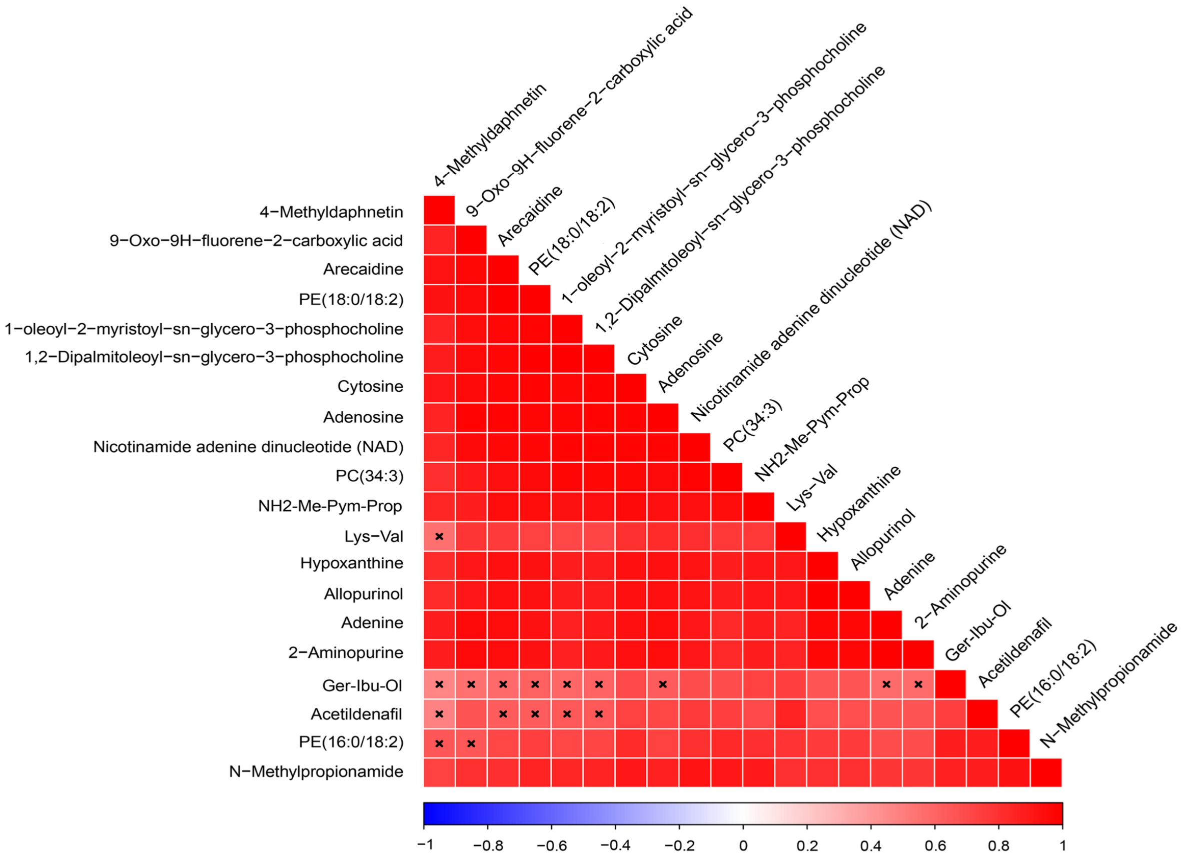

e

Figure S3. Correlation analysis of differential metabolites in P vs. Control. NEG mode at day 0 (a) and POS mode (b); POS mode at day 1 (c); NEG mode at day 3 (d) and POS mode (e). Symbols marked with “×” indicate correlations with |r| < 0.6.

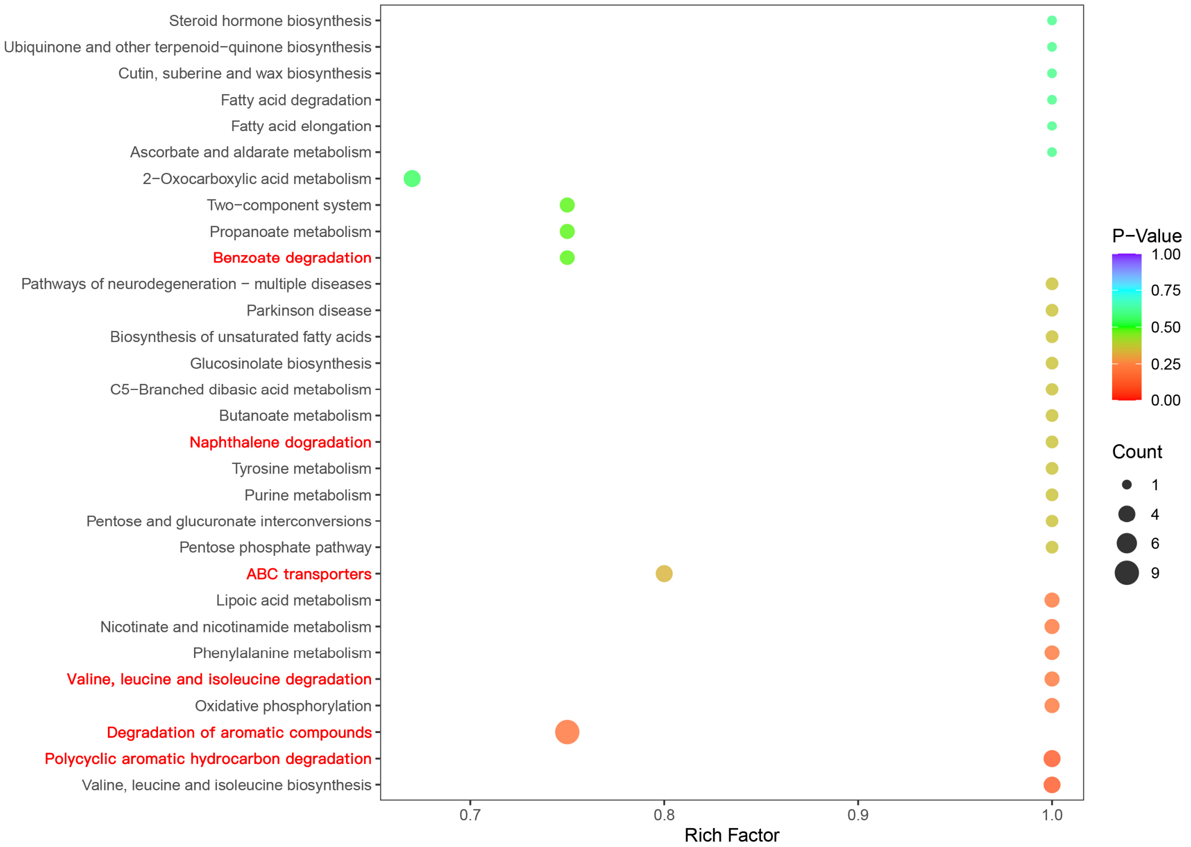
Steroid hormone biosynthesis
Ubiquinone and other terpenoid−quinone biosynthesis
Cutin, suberine and wax biosynthesis
Fatty acid degradation
Fatty acid elongation
Ascorbate and aldarate metabolism
2−Oxocarboxylic acid metabolism
Two−component system
Propanoate metabolism
Benzoate degradation
Pathways of neurodegeneration − multiple diseases
Parkinson disease
Biosynthesis of unsaturated fatty acids
Glucosinolate biosynthesis
C5−Branched dibasic acid metabolism
Butanoate metabolism
Naphthalene dogradation
Tyrosine metabolism
Purine metabolism
Pentose and glucuronate interconversions
Pentose phosphate pathway
ABC transporters
Lipoic acid metabolism
Nicotinate and nicotinamide metabolism
Phenylalanine metabolism
Valine, leucine and isoleucine degradation
Oxidative phosphorylation
Degradation of aromatic compounds
Polycyclic aromatic hydrocarbon degradation
Valine, leucine and isoleucine biosynthesis
0.7
0.8
0.9
1.0
Rich Factor
P−Value
1.00
0.75
0.50
0.25
0.00
Count
1
4
6
9

a

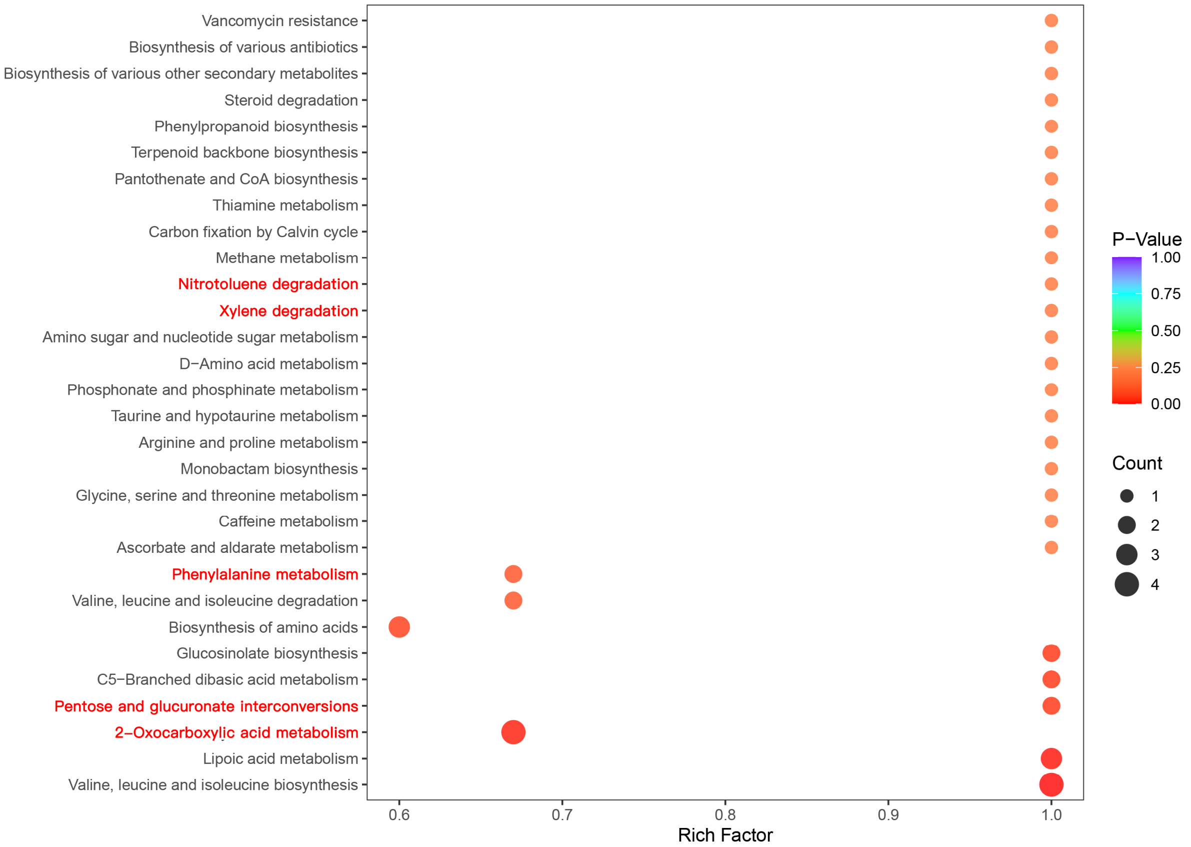
Vancomycin resistance
Biosynthesis of various antibiotics
Biosynthesis of various other secondary metabolites
Steroid degradation
Phenylpropanoid biosynthesis
Terpenoid backbone biosynthesis
Pantothenate and CoA biosynthesis
Thiamine metabolism
Carbon fixation by Calvin cycle
Methane metabolism
Nitrotoluene degradation
Xylene degradation
Amino sugar and nucleotide sugar metabolism
D−Amino acid metabolism
Phosphonate and phosphinate metabolism
Taurine and hypotaurine metabolism
Arginine and proline metabolism
Monobactam biosynthesis
Glycine, serine and threonine metabolism
Caffeine metabolism
Ascorbate and aldarate metabolism
Phenylalanine metabolism
Valine, leucine and isoleucine degradation
Biosynthesis of amino acids
Glucosinolate biosynthesis
C5−Branched dibasic acid metabolism
Pentose and glucuronate interconversions
2−Oxocarboxylic acid metabolism
Lipoic acid metabolism
Valine, leucine and isoleucine biosynthesis
0.6
0.7
0.8
0.9
1.0
Rich Factor
P−Value
1.00
0.75
0.50
0.25
0.00
Count
1
2
3
4

b

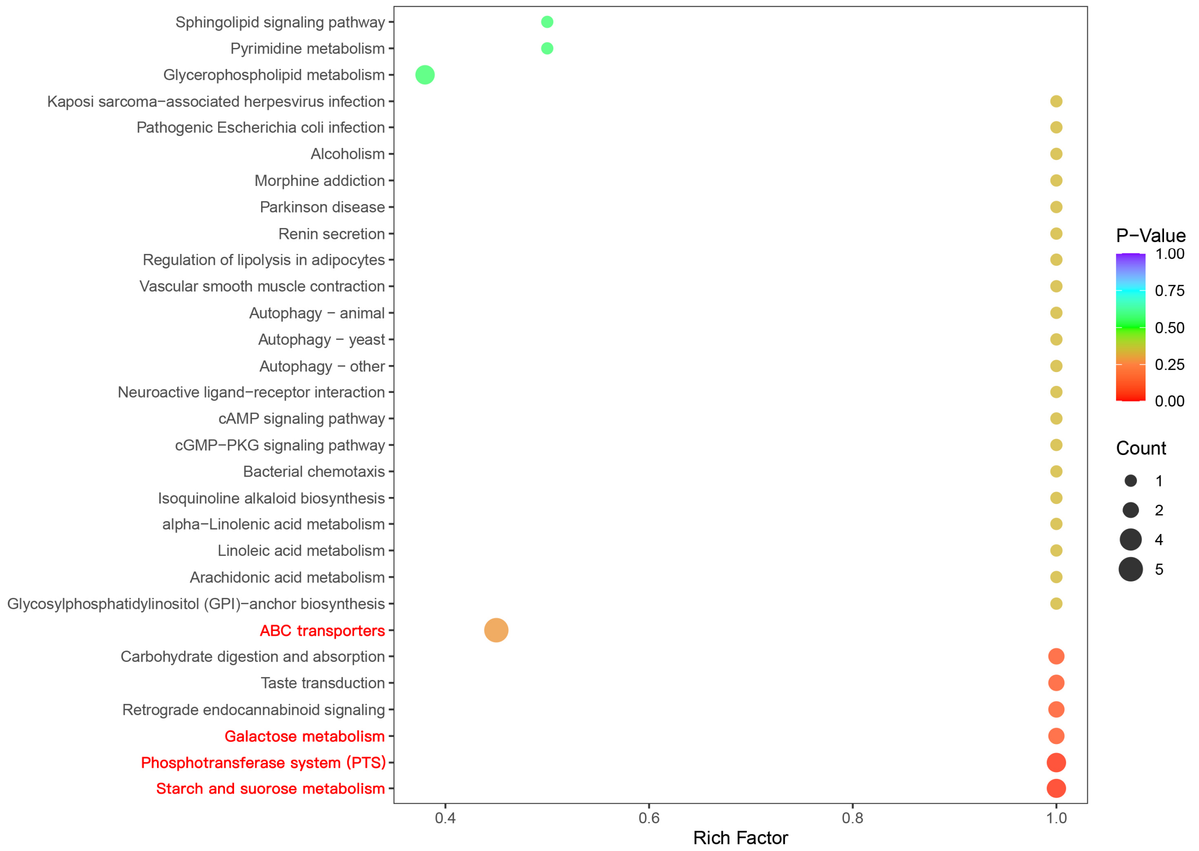
Sphingolipid signaling pathway
Pyrimidine metabolism
Glycerophospholipid metabolism
Kaposi sarcoma−associated herpesvirus infection
Pathogenic Escherichia coli infection
Alcoholism
Morphine addiction
Parkinson disease
Renin secretion
Regulation of lipolysis in adipocytes
Vascular smooth muscle contraction
Autophagy − animal
Autophagy − yeast
Autophagy − other
Neuroactive ligand−receptor interaction
cAMP signaling pathway
cGMP−PKG signaling pathway
Bacterial chemotaxis
Isoquinoline alkaloid biosynthesis
alpha−Linolenic acid metabolism
Linoleic acid metabolism
Arachidonic acid metabolism
Glycosylphosphatidylinositol (GPI)−anchor biosynthesis
ABC transporters
Carbohydrate digestion and absorption
Taste transduction
Retrograde endocannabinoid signaling
Galactose metabolism
Phosphotransferase system (PTS)
Starch and suorose metabolism
0.4
0.6
0.8
1.0
Rich Factor
P−Value
1.00
0.75
0.50
0.25
0.00
Count
1
2
4
5

c

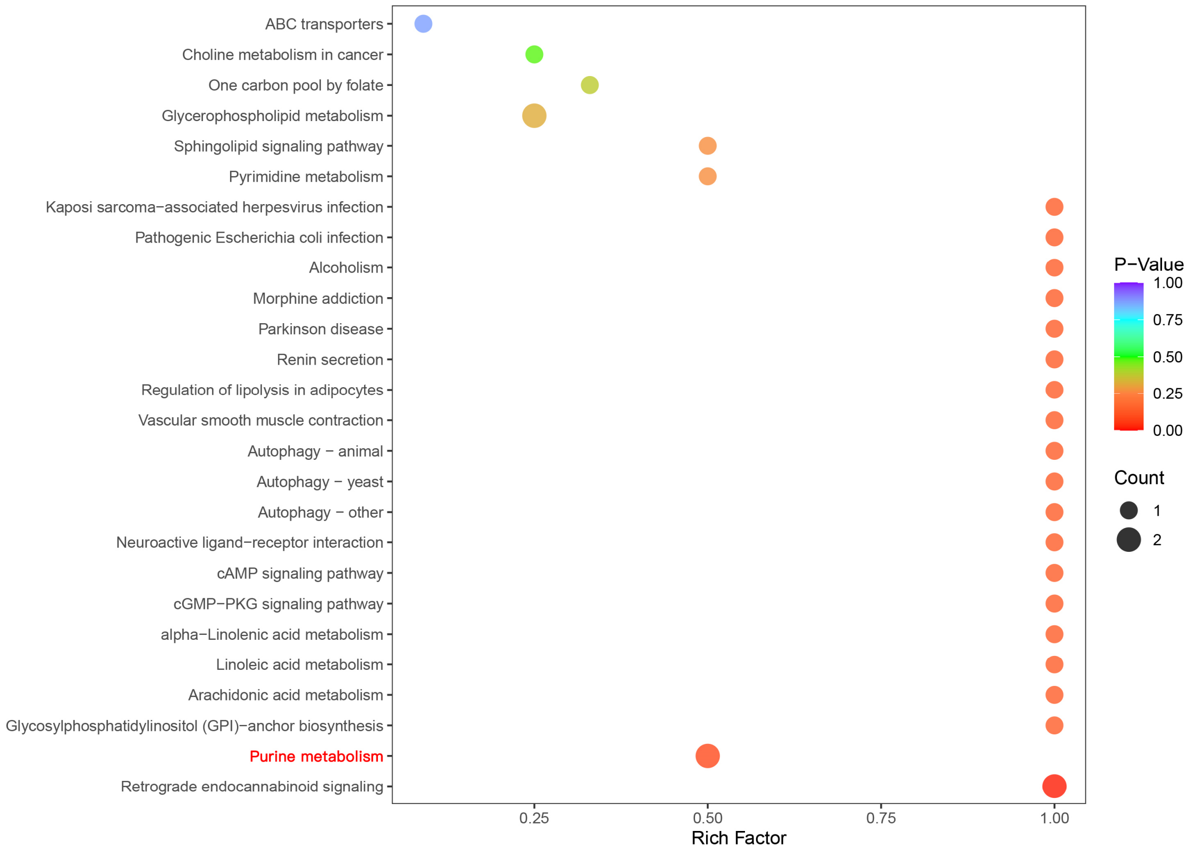
ABC transporters
Choline metabolism in cancer
One carbon pool by folate
Glycerophospholipid metabolism
Sphingolipid signaling pathway
Pyrimidine metabolism
Kaposi sarcoma−associated herpesvirus infection
Pathogenic Escherichia coli infection
Alcoholism
Morphine addiction
Parkinson disease
Renin secretion
Regulation of lipolysis in adipocytes
Vascular smooth muscle contraction
Autophagy − animal
Autophagy − yeast
Autophagy − other
Neuroactive ligand−receptor interaction
cAMP signaling pathway
cGMP−PKG signaling pathway
alpha−Linolenic acid metabolism
Linoleic acid metabolism
Arachidonic acid metabolism
Glycosylphosphatidylinositol (GPI)−anchor biosynthesis
Purine metabolism
Retrograde endocannabinoid signaling
0.25
0.50
0.75
1.00
Rich Factor
P−Value
1.00
0.75
0.50
0.25
0.00
Count
1
2

d

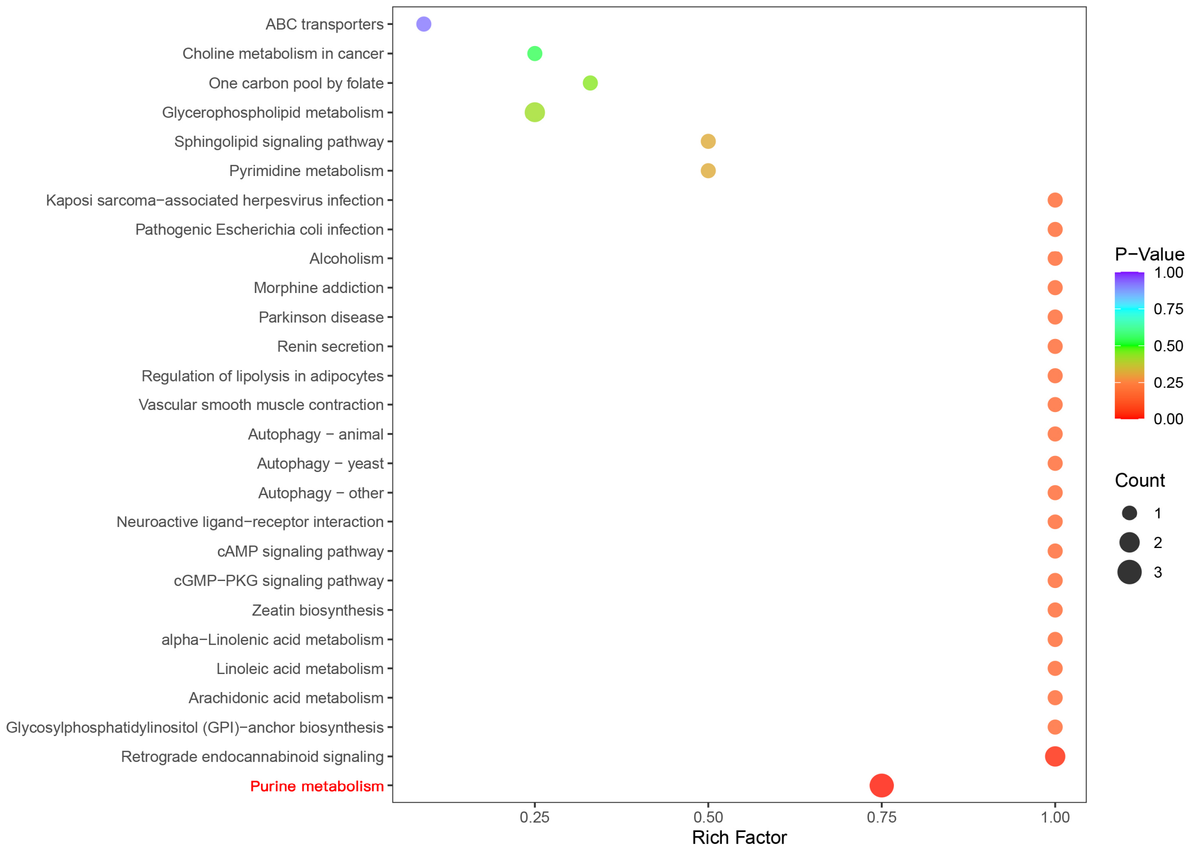

e

Figure S4 KEGG enrichment plot of differential metabolites between P group and control. NEG mode at day 0 (a) and POS mode (b); POS mode at day 1 (c); NEG mode at day 3 (d) and POS mode (e). Metabolites marked in red indicate the key substances mentioned in the text.
